# A new thinking: deciphering the aberrance and clinical implication of copper-death signatures in clear cell renal cell carcinoma

**DOI:** 10.1186/s13578-022-00948-7

**Published:** 2022-12-29

**Authors:** Aimin Jiang, Peng Luo, Ming Chen, Yu Fang, Bing Liu, Zhenjie Wu, Le Qu, Anbang Wang, Linhui Wang, Chen Cai

**Affiliations:** 1 Department of Urology, Changhai Hospital, Naval Medical University (Second Military Medical University), Shanghai, 200433 China; 2 Department of Oncology, Zhujiang Hospital, Southern Medical University, Guangzhou, 510280 China; 3 Department of Urology, Changzheng Hospital, Naval Medical University (Second Military Medical University), Shanghai, 200003 China; 4 Department of Urology, The Third Affiliated Hospital, Naval Medical University (Second Military Medical University), Shanghai, 201805 China; 5 Department of Urology, Affiliated Jinling Hospital, Medical School of Nanjing University, Nanjing, 210046 China; 6 Department of Special Clinic, Changhai Hospital, Naval Medical University (Second Military Medical University), Shanghai, 200433 China

**Keywords:** Copper induced cell death, Pan-cancer, Clear cell renal cell carcinoma, Molecular subtypes, Tumor immunity

## Abstract

**Rationale:**

Recent research has indicated that cuprotosis, or copper induced cell death, is a novel type of cell death that could be utilized as a new weapon for cancer management. However, the characteristics and implications of such signatures in cancers, especially in clear cell renal cell cancer (ccRCC), remain elusive.

**Methods:**

Expression, methylation, mutation, clinical information, copy number variation, functional implication, and drug sensitivity data at the pan-cancer level were collected from The Cancer Genome Atlas. An unsupervised clustering algorithm was applied to decipher ccRCC heterogeneity. Immune microenvironment construction, immune therapy response, metabolic pattern, and cancer progression signature between subgroups were also investigated.

**Results:**

Cuprotosis related genes were specifically downregulated in various cancer tissues compared with normal tissues and were correlated with hypermethylation and copy number variation. Cuprotosis scores were also dysregulated in tumor tissues, and we found that such a signature could positively regulate oxidative phosphorylation and Myc and negatively regulate epithelial mesenchymal translation and myogenesis pathways. CPCS1 (cuprotosis scores high) and CPCS2 (cuprotosis scores low) in ccRCC displayed distinctive clinical profiles and biological characteristics; the CPCS2 subtype had a higher clinical stage and a worse prognosis and might positively regulate cornification and epidermal cell differentiation to fuel cancer progression. CPCS2 also displayed a higher tumor mutation burden and low tumor stemness index, while it led to a low ICI therapy response and dysfunctional tumor immunity state. The genome-copy numbers of CPCS2, including arm- gain and arm- loss, were higher than those of CPCS1. The prognostic model constructed based on subgroup biomarkers exerted satisfactory performance in both the training and validation cohorts. In addition, overexpression of the copper death activator DLAT suppressed the malignant ability, including cell migration and proliferation, of renal cell lines in vitro and in vivo. Finally, activation of cuprotosis in tumors could enhance antitumor immunity through dsDNA-cGAS-STING signaling in ccRCC.

**Conclusion:**

The activation of cuprotosis might function as a promising approach among multiple cancers. The cuprotosis related signatures could reshape tumor immunity in the ccRCC microenvironment via cGAS-STING signal, thus activating tumor antigen-presenting process. Upregulation of DLAT expression in ccRCC cell lines could reactivate the copper death pattern and be treated as a suitable target for ccRCC.

**Supplementary Information:**

The online version contains supplementary material available at 10.1186/s13578-022-00948-7.

## Introduction

Even equipped with advanced diagnostic and therapeutic approaches, cancer is still the leading disease that accounts for approximately 15% of all deaths worldwide [1]. With the prolongation of human lifespan, the incidence and mortality of cancers continue to increase. Considering the heterogeneity and homogeneity of tumorigenesis, it is extremely significant to explore and evaluate novel prognostic or therapeutic targets, which could function as a paradigm in the systematic management era. Renal cancer is a common urinary tract tumor with insidious onset and the most common type is clear cell renal cell carcinoma (ccRCC). Early-stage renal cancer has no obvious symptoms. Approximately 20%-30% of patients have metastasized at the first visit, and nearly 30% of renal cancer patients suffer to recurrence and metastasis after surgery [2, 3]. However, advanced metastatic renal cancer is not sensitive to radiotherapy and chemotherapy, so the clinical treatment for those patients is extremely limited, and the prognosis is very poor, with a 5-year survival rate of less than 5% [4]. TKI-targeted drugs and immune checkpoint inhibitors that have emerged in the past decade have brought some hope to ccRCC patients. However, current treatments lack specificity in the treatment of advanced renal cancer, with large toxicity and side effects, frequent drug resistance, and median survival of patients less than 3 years. Thus, the combination of drug therapy might a be proper approach to cure renal cancer. The European Association of Urology Guidelines advise immunotherapy combined with targeted therapy as the first-line treatment of ccRCC. Lisa et al. recorded that gut bacterial composition derived primary resistance to cancer immunotherapy in renal cell carcinoma patients. Homeostasis of the gut microbiota may help to attenuate immunotherapy resistance [5]. Accordingly, combination therapies might be promising directions for ccRCC treatment. It is compelling to develop new targets or assistant targets based on molecular features.

Copper induced cell death is a newly discovered type of cell death that is different from apoptosis, ferroptosis, pyroptosis and necroptosis. Copper is an important enzymatic cofactor in cellular processes, but even modest intracellular concentrations could be toxic, leading to cell death. It has been recognized that the demand for copper is elevated in tumor cells relative to most other tissues, and represents a metabolic vulnerability that can be exploited by limiting copper availability [6]. Genetic variations in copper homeostasis cause life-threatening diseases, and both copper ionophores and copper chelators are considered anticancer agents. Copper can induce multiple forms of cell death through various mechanisms, including reactive oxygen species accumulation, proteasome inhibition, and anti-angiogenesis. The introduction of copper in vivo has become a research hotspot in the field of tumor therapy. Lu et al. showed that copper-based Mcl-1 inhibitors can be used to treat Mcl-1-related cancers with high efficiency and low toxicity [7]. Zhang et al. found that copper oxide nanoparticle exposure leads to mitochondrial dysfunction and the accumulation of mitochondrial superoxide anions in HUVECs, mediating p38 MAPK activation and inducing DNA oxidative damage and cell death [8]. More recently, Peter et al. explored and demonstrated the specific mechanism of copper induced cell death [9]. They observed that cuproptosis was dependent on mitochondrial respiration and that cuproptosis occurs through the direct binding of copper to the fatty acylated component of the tricarboxylic acid (TCA) cycle. This leads to fatty acylated protein aggregation and subsequent loss of iron-sulfur cluster proteins, causing proteotoxic stress and ultimately cell death. Therefore, copper ionophore therapy could target cancers with high levels of fatty acylated proteins. It is believed that in the future, the use of copper ion metal carriers to kill cancer cells will become a new method for cancer treatment.

In this study, we conducted a characterization study of cuproptosis related genes by pan-cancer analysis and stratified ccRCC patients by integrating multiomics data, including prognostic analysis, gene mutation analysis, immune infiltration analysis, and drug sensitivity analysis, and constructed a reliable risk stratification model to predict the prognosis of ccRCC patients. In addition, we identified and confirmed a promising cuproptosis target DLAT, which could function as a new therapeutic target for ccRCC.

## Materials and method

### Data collection and processing

The workflow of this study is depicted in Additional file 1: Fig. S1. Pan-cancer normalized expression profiling data, DNA methylation data, tumor mutation burden (TMB), microsatellite instability (MSI), copy number variation (CNV), somatic mutation data and clinical characteristics were downloaded from the UCSC XENA dataset (http://xena.ucsc.edu/) [10]. The external ccRCC cohort, E-MTAB-1980, or JAPAN-KIRC which included expression profile and prognostic information, was downloaded from Express-array database (https://www.ebi.ac.uk/arrayexpress/), and different stage single cell sequence data of ccRCC patients were collected from GEO (ID PRJNA705464; https://www.ncbi.nlm.nih.gov/geo/). Two ccRCC single cell sequence datasets, including GSE159115 and GSE171306, were also adopted. This study also utilized several public cancer datasets, including UALCAN (http://ualcan.path.uab.edu/index.html), TIMER (https://cistrome.shinyapps.io/timer/), TIDE (http://tide.dfci.harvard.edu/), and MEXPRESS (https://mexpress.be/) [11]. Ethical Review Committee approval and informed consent were not required for datasets downloaded from public datasets. Patients without prognostic information or expression profiles or who died within 30 days were excluded from this study.

### Identification of distinct cuproptosis subgroups in ccRCC

We collected 10 cuproptosis related genes according to Tsvetkov et al. Additional file 2: Table S1 R package corrplot was used to calculate the correlation among these genes via Spearman and Pearson rank algorithms. Consensus clustering was performed according to the expression matrix of cuproptosis related genes via R package ConsensusClusterPlus [12]. The subtype number k = 2 was determined as the best classification number.

### Enrichment analysis between subgroups

R package DEseq2 was used to identify differentially expressed genes (DEGs) between subgroups; thresholds were set at adjusted p < 0.01, and the abstract log Foldchange > 2. After calculating the DEGs, R package ClusterProfiler was used to perform Gene Ontology (GO), Kyoto Encyclopedia of Genes and Genomes (KEGG) pathways, Gene Set Enrichment Analysis (GSEA) and Gene Set Variation Analysis (GEVA), aiming to explain the biological function and molecular mechanism between CPCS1 and CPCS2. All gmt files used for enrichment analysis were downloaded from the MSigDB (https://www.gsea-msigdb.org/gsea/index.jsp) and the ConsensusPathDB (http://cpdb.molgen.mpg.de/) database [13–15].

### Differences in immune infiltration signatures and therapy response

We utilized multiple immune cell infiltration algorithms including TIMER, CIBERSORT, QUANTISEQ, MCPCOUNTER, XCELL, and EPIC to calculate cellular components or immune cell enrichment scores in ccRCC tissues [16–19]. In addition, single-sample gene set enrichment analysis (ssGSVA) was introduced to further validate such differences [20]. R package ESTIMATE was used to evaluate the stromal and immune scores based-on transcriptome profiling. Tumor Immune Dysfunction and Exclusion (TIDE, http://tide.dfci.harvard.edu/) algorithm was used to compare immunotherapy responses between subgroups [21].

### Mutation spectrum characteristics between subpopulations

Somatic data were analyzed and visualized via R package Maftools to compare mutational patterns between subgroups [22]. Through the transformation analysis function module, the drug and gene interactions and the differences in oncogenic signaling pathways of different subsets were also analyzed. Analysis of recurrent extensive and focal somatic copy number alterations (SCNA) was performed by the GISTIC 2.0 (https://cloud.genepattern.org/gp/pages/index.jsf) algorithm based on Euclidean distance [23, 24].

### Drug susceptibility prediction

Each patient was assessed for their susceptibility to molecular drugs using the Genomics of Cancer Drug Sensitivity (GDSC, https://www.cancerrxgene.org/) database. R package pRRophetic was utilized to estimate the half-maximal inhibitory concentration-IC50. In addition, CellMiner (https://discover.nci.nih.gov/cellminer/home.do) and CCLE (https://sites.broadinstitute.org/ccle) databases were also introduced to compare the different sensitivities between ccRCC cell lines [25–27]. Spearman's correlation coefficient was used to identify whether gene expression was associated with drug sensitivity. A positive correlation means that high expression of the gene indicates resistance to the drug, and low expression indicates sensitivity to such therapy.

### Construction of a risk prediction model related to cuproptosis

First, using subgroup-related biomarkers and overall prognostic information from the TCGA-KIRC cohort, univariate Cox regression analysis was performed to select survival-related signatures. Then, random survival forest variable hunting (RSFVH) algorithm was further performed to select crucial signatures. Finally, a risk scoring model was constructed using the best combination of prognostic genes to screen. The JAPAN-ccRCC cohort was used to validate our risk scoring model, and patients in both datasets were divided into high- and low-risk groups based on median risk scores.

### Western blotting, IHC and RT-qPCR

RT-qPCR and immunohistochemical (IHC) staining were performed to validate DLAT expression in paired tumor and adjacent renal tissue (including 40 clear cell renal carcinoma tissues from Changhai Hospital). Primer sequences for RT-qPCR were as follows: primer for DLAT (forward: CGGAACTCCACGAGTGACC, reverse: CCCCGCCATACCCTGTAGT), STING(forward: TCGCACGAACTTGGACTACTG, reverse: CCAACTGAGGTATATGTCAGCAG), TBK1(forward: GGAGCCGTCCAATGCGTAT, reverse: GCCGTTCTCTCGGAGATGATTC), cGAS(forward TTCCACGAGGAAATCCGCTGAG, reverse: CAGCAGGGCTTCCTGGTTTTTC), IRF2(forward GAGAGCCGAACGAGGTTCAG, reverse: CTTCCAGGTTGACACGTCCG) and GAPDH (forward: GGAGCGAGATCCCTCCAAAAT, reverse primer: GGCTGTTGTCATACTTCTCATGG). Antibodies of DLAT (Cat no: 13426–1-AP), cGAS (Cat no: ab252416) and GAPDH (Cat no: ab8245) were purchased from ProteinTech and Abcam Group. Ltd. The detailed procedure referred to our previous researcher’s protocols [28–30].

### Investigation of DLAT biological function in vitro and in vivo

All the cell lines used in our study, including human and mouse normal renal epithelial cell lines HK-2 and CP-M062 and cancer cell lines (including 769-P, 786-O, A-498, Caki-1, Caki-2, OSRC-2 and RENCA), were purchased from the American Type Culture Collection (ATCC). Cell lines were cultured according to the instructions. The DLAT overexpression plasmid was chemically synthesized by Shanghai GeneChem Co., Ltd. 786–0 and OSRC-2 cells were cultured in 6-well culture dishes at 60% density and then infected with DLAT overexpression lentivirus and the negative control (NC) lentivirus. All transfections were supplied with 4ug/ml polybrene (H8761; Solarbio, Inc) and lasted for 12 h. Screening was conducted with 2ug/ml puromycin (P8833; Sigma, Inc) for 3 days to acquire stably transfected cells. QT-PCR and Western blotting were applied to verify the overexpression efficiency of the lentivirus. CCK-8 (Cell Counting Kit-8) was used to detect the difference in cell viability between the negative control and DLAT-overexpression groups. Scratch assays and Transwell migration assays were used to evaluate cell migration ability. A colony-forming experiment was conducted to determine the reproductive ability in vitro. A nude mouse xenograft tumor model assay was applied to investigate the effect of DLAT overexpression in vivo. Nude mice were given xenografts of DLAT overexpression and control 786-O cells (5 × 10^6^ cells per site). The tumors were dissected and photographed after approximately 4 weeks (n = 5 per group). The tumors were dissected and photographed after approximately 4 weeks (n = 5 per group). Tumor volumes were measured after tumor resection. The detailed procedure of the in vivo experiments is described in our previous study [31].

### Impact of cuprotosis in ccRCC tumor immunity

Cuprotosis reagents, Elesclomol and Cucl2, were obtained from MedChemExpress. Immune check inhibitor agent (anti-PD-1) was purchased from BioXcell (Clone RMP1–14). ELISA kits for mouse IL6, TNF-α, IFN-γ, CXCL10 and CXCL11, and cytotoxicity assays based lactate dehydrogenase (LDH) release were purchased form Thermofisher. The cGAMP Activity Assay Kit was obtained from BellBrook. Tumor cell growth was evaluated via IVIS at 12 and 24 days after tumor cell injection. The antibodies for fluorescent dye-conjugated of flow cytometry, including CD45-BV510 and CD8-APC-Cy7, CD3-PE-Cy7, were purchased from Biolegend and eBioscience. The detailed procedure of intracellular staining can be found in each protocol. Flow cytometry analysis was performed with a FACS LSRII or Fortessa X-20 (BD Biosciences, San Jose, CA, USA) and data were analyzed by FlowJo v.10 software (FlowJo, Ashland, OR, USA).

### Co-culture system

The optimal concentration of cuprotosis inducer reagents (Elesclomol + Cucl2) for renal cancer cell lines were identified by LDH release assay results between CP-M062 and RENCA. Dendritic cells, or DCs, were a kind gift from Department of Immunology (National Key Laboratory of Medical Immunology, Naval Medical University). In detail, DCs were derived from CD14^+^ monocytes from health donor’s peripheral venous blood, which were cultured in RPMI-1650 (containing 10%FBS, GM-CSF(50 ng/ml) and IL-4(10 ng/ml)), then stimulated by lipopolysaccharide, or LPS(1ug/ml). Before co-culture, ACHN were treated with DMSO or cuprotosis inducer reagents (Elesclomol + Cucl2) at concentration of 2 μmol/L for 3 days. Then DCs were co-cultured with tumor cells pre-treated with DMSO or cuprotosis inducer reagents at a 5:1 ration in the presence of IL-4 (10 ng/ml). After 2 days co-culture, DCs were harvested to perform Western blotting, qPCR, and ELISA to quantify different level of cGAS-STING signal and immune related molecules.

### Mouse subcutaneous xenograft model

Athymic nude mice (BALB/c, 4–6-week-old) and C57BL/6 mice (5–6-week-old) were obtained from Model Organisms (Shanghai, China). Renal cancer cells, including ACHN and RENCA treated with DMSO, ICI, or cuprotosis inducer reagents (Elesclomol + Cucl2), were injected into right flank of mice (1.0 × 10^6^ cells per mice), respectively. BALB/c mice inoculated with RENCA were observed after 12 days; then those mice were randomized divided into three groups receiving DMSO, cuprotosis inducer reagents at concentration of 2 and 5 μmol/L, and tumor was identified at 24 days by bioluminescent imaging by IVIS Lumina K series III (PerkinElmer Inc, Hopkinton, MA, USA). After the xenografts reached a size of approximately 45 mm^3^, C57BL/6 mice were randomized into four groups receiving DMSO, anti-PD-1mAB, cuprotosis inducer reagents, and combined therapy (anti-PD-1 + cuprotosis inducer reagents) by intraperitoneal (i.p., given at days 14, 17, 20), respectively. After one week, all mice treated with those agents were euthanized and peripheral blood were harvested to quantity the percentage of CD45 + CD8 + T cells among different groups by flow cytometry. All those procedures were approved by the Institutional Animal Care and Use Committee at Navel Medical University.

### Statistical analysis

All data processing, statistical analysis and plotting were performed via R software (version 4.0.4). Differences between subgroups were compared by Kruskal–Wallis test and Wilcoxon test. Differences in clinical characteristics and inhibitor response between subgroups were compared by the chi-square test. Differences in prognosis, including overall survival (OS) and progression-free survival (PFS) were compared by Kaplan–Meier method and Log-rank test. Hazard ratio (HR) differences were calculated by univariate Cox regression and multiple Cox regression analyses. Two-way p-values were taken and P < 0.05 was considered statistically significant.

## Results

### Dysregulation and mutation of copper induced cell death related genes in cancers

**Fig. 1:**
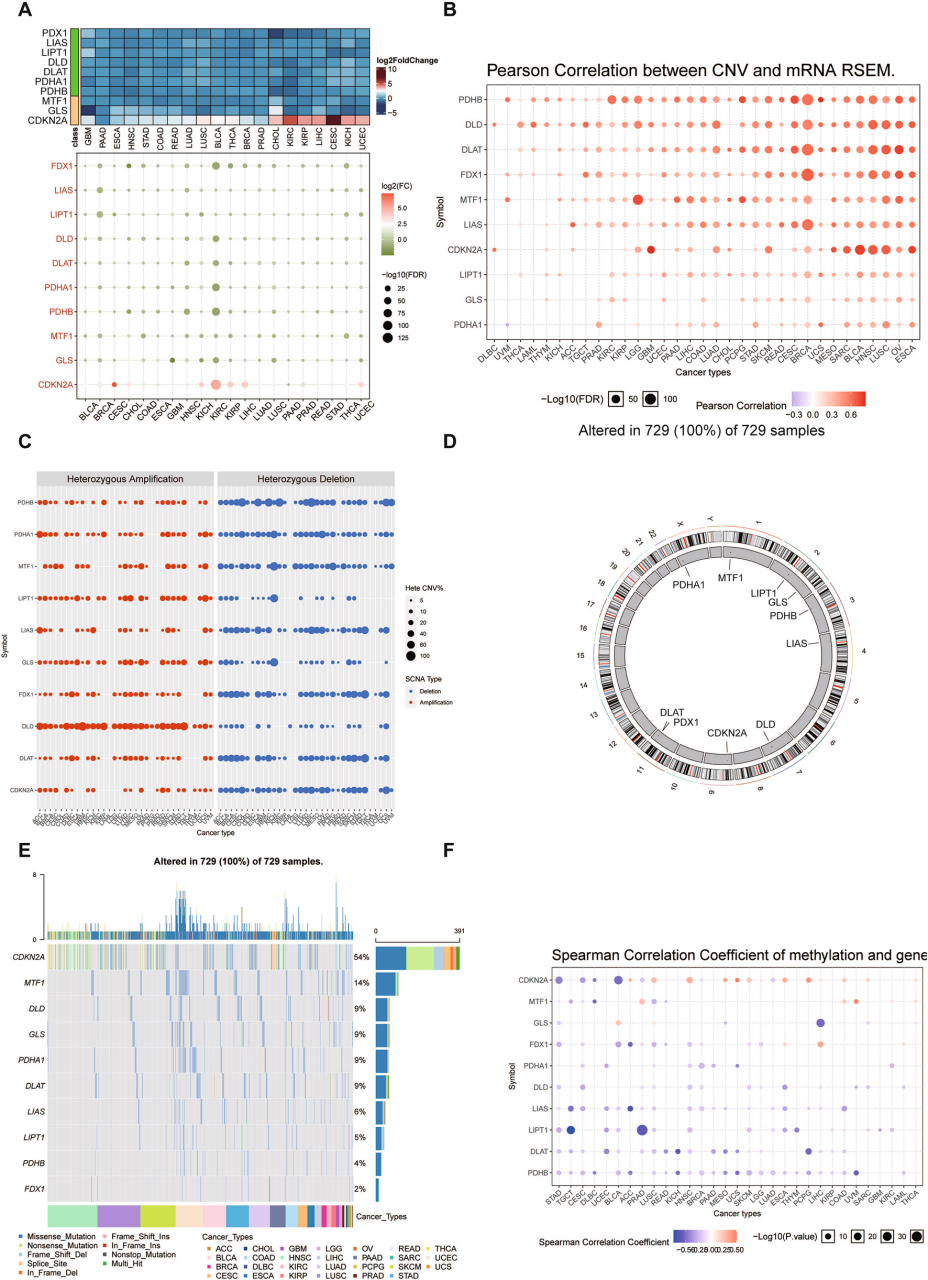
Dysregulation and mutation of copper-induced cell death-related genes in cancers. **A** Gene expression of copper-induced cell death-related genes between multiple cancer tissues and normal tissues. **B** Correlation analysis of CNV with the gene expression of copper-induced cell death-related genes. **C** Heterozygous and homozygous amplification/deletion of copper induced cell death-related genes in multiple cancers. **D** The genome locations of copper-induced cell death-related genes on 23 chromosomes. **E** Mutation type of copper induced cell death-related genes in multiple cancers. **F** The methylation analysis of CNV with gene expression of copper-induced cell death-related genes

**Fig. 2:**
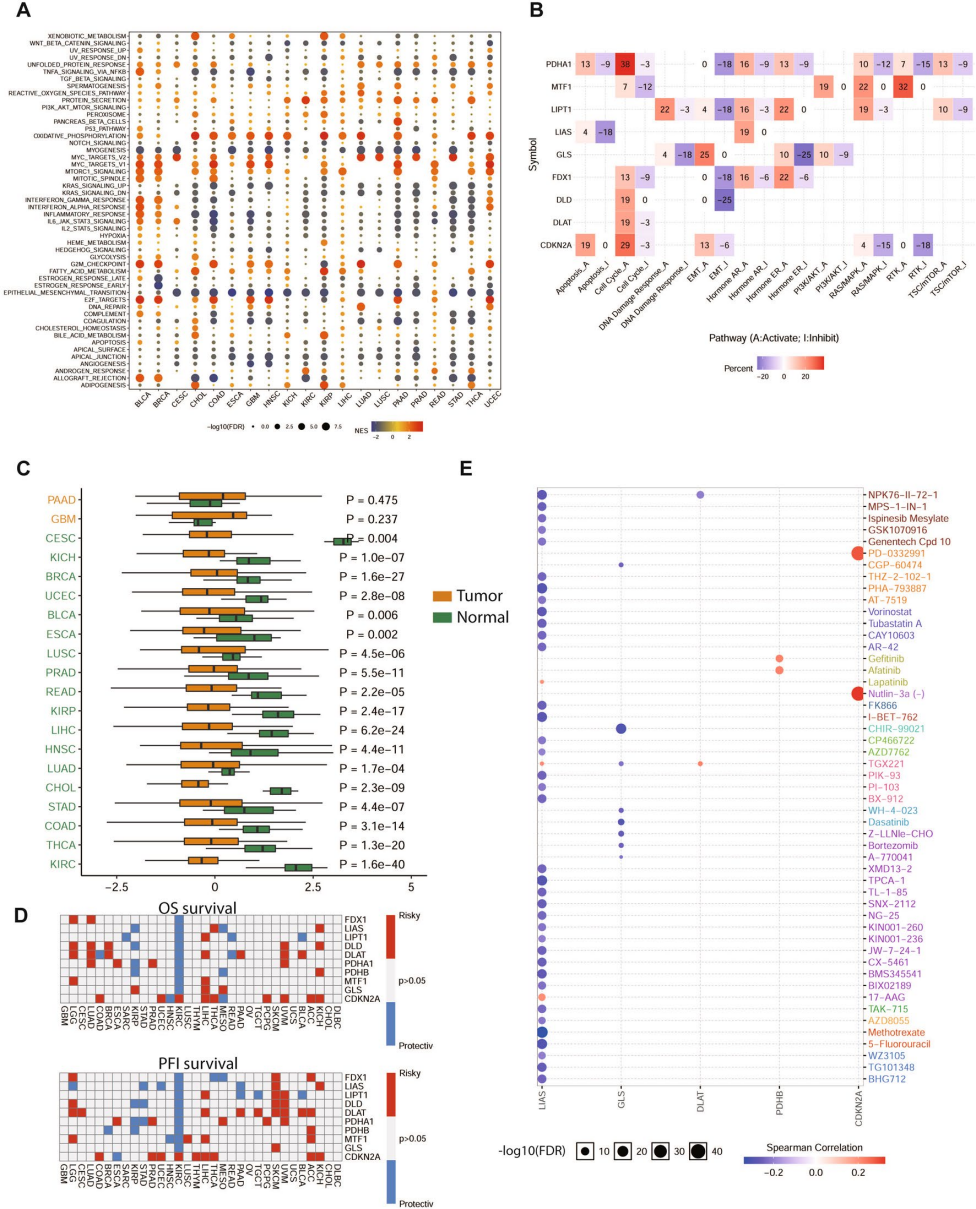
Pathway enrichment analysis and drug sensitivity assessment of copper-induced cell death genes. **A** Heatmap of multiple cancer-related pathways for copper-induced cell death. **B** The activation of the inhibition pathway in multiple cancers. **C** The copper-induced death potential index (CPI) in cancer and normal tissues. **D** The association between the expression levels of copper-induced cell death-related genes and patient outcomes. **E** The drug sensitivity assessment of several copper-induced cell death-related genes to molecular inhibitors

Copper induced cell death has gradually become the focus of attention for cancer research. We first explored the expression pattern of copper induced cell death related genes across cancers. As shown in Fig. 1A, most copper induced cell death genes were downregulated in various cancers, such as FDX1 in cholangiocarcinoma (CHOL); LIAS and LIPT1 in bladder cancer (BLCA); PDHB in glioblastoma (GBM) and kidney chromophobe cancer (KICH). The results demonstrated that copper induced death was suppressed across cancers. Nonetheless, CDKN2A expression was significantly up regulated KICH and breast cancer (BRCA). The classic regulator of copper induced death FDX1 was investigated and was significantly reduced in renal cancer, liver cancer, gastric adenocarcinoma, and other cancers compared with the normal control (Additional file 1: Fig. S2A). To comprehensively understand the dysregulated expression of copper induced cell death genes, we investigated the characteristics of copy number variation (CNV) and single-nucleotide variation (SNV) across cancers (Fig. 1B). In most cancers, CNV and gene expression were significantly correlated, especially in BRCA, BLCA, HNSC, LUSC and OV. As depicted in Fig. 1C, heterozygous amplifications frequently appeared in DLD, PHDA1, and LIPT1, while heterozygous deletions often occurred in PHDB, FDX1, LIAS, and DLAT. Figure 1D shows the genomic location of copper induced cell death genes, which were distributed on several chromosomes. The SNV frequency of copper induced cell death genes was analyzed, and all 729 samples tested had at least one mutation site (Fig. 1E). CDKN2A, MTF1, DLD, GLS, PDHA1 and DLAT exhibited higher mutation frequencies, and the SNV rate of CDKN2A even exceeded 50%. Cancers with high rates of SNVs included HNSC, LUSC, PAAD, BLCA, LUAD, SKCM and UCEC (Additional file 1: Fig. S2B). Methylation is usually negatively correlated with gene expression. Likewise, the expression of most copper induced cell death genes was negatively correlated with their methylation status, such as PHDB, DLAT, LIPT1 and LIAS (Fig. 2F). Thus, most of the copper induced cell death genes were hypermethylated states in cancers. CDKN2A expression was positively related to methylation levels in HNSC, ESCA, PCPG and LUSC. MTF1 expression in PRAD, COAD, UVM and FDX1 expression in LIHC exhibited positive correlation with methylation status. Hypermethylated PDHB, LIPT1, LIAS and GLS were mainly associated with poor prognosis in cancer, whereas hypomethylated FDX1 was dominantly related to poor prognosis of LGG and ccRCC (Additional file 1: Fig. S2C). Therefore, copy number variation, single nucleotide variation and methylation status together contributed to the dysregulation of copper induced cell death in pan-cancer.

### Pathway enrichment analysis and drug sensitivity assessment of copper induced cell death genes

After exploring the fundamental reasons for copper induced cell death gene dysregulation, we analyzed the effect of these genes on cancer signaling pathways. GSEA analyses indicated that copper induced cell death genes were mainly positively related to the MYC, MTOR, G2M checkpoint and oxidative phosphorylation signaling pathways, but negatively correlated with epithelial mesenchymal transition (EMT), myogenesis and TNFA—NFκB (Fig. 2A). Specifically, PDHA1 was associated with cell cycle, hormone AR pathway activation, EMT inhibition and RTK inhibition. LIPT1 was associated with DNA damage response, hormone AR activation, and EMT inhibition. LIAS was enriched in hormone AR activation and apoptosis inhibition. FDX1 was mainly enriched in hormone AR, ER activation and EMT inhibition. MTF1 was related to RTK pathway, RAS-MAPK pathway activation and cell cycle inhibition. CDKN2A was associated with cell cycle activation and RTK, RAS-MAPK inhibition (Fig. 2B). The positive factors related to copper induced death were mainly enriched in cell cycle, metabolism, RAS-MAPK activation and EMT inhibition pathways.

Considering the importance of copper induced death, we determined copper induced death potential index (CPI), similar to ferroptosis in cancer and normal tissues [32]. CPI index was significantly lower in most cancers than in normal tissues, especially in ccRCC, breast cancer and liver cancer (Fig. 2C). In addition, we found that high expression of most of these genes predicted poor prognosis in cancer patients (Fig. 2D), such as FDX1 in LUAD and LGG; LITP1 in SKCM and UVM; DLAT in BLCA and LIHC; and CDKN2A in KICH, ACC, THCA, LIHC, LIHC, KIRC UCEC and COAD. Notably, most genes except CDKN2A indicated a protective function for ccRCC patients. The GDSC and CTRP databases were used to perform drug sensitivity analysis of copper induced cell death-related genes. Spearman correlation analysis showed that high CDKN2A expression exhibited good sensitivity to PD-0332991 and Nutlin-3a. Similar results were found for PDHB to Gefitinib and Afatinib (Fig. 2E). GLS was responsible for GSK-J4 and tivantinib sensitivity (Additional file 1: Fig. S3). LIAS was associated with multiple drug resistance, such as Methotrexate and TPCA-1. GLS was closely related to CHIR-99021, dasatinib and bortezomib resistance. These drug sensitivity results may be developed to find effective targets for cancer treatment. LIA and GLS expression might be responsible for therapy resistance, which needs further research.

### Establishment of two clusters by clustering analysis of copper induced cell death-related genes in ccRCC

**Fig. 3:**
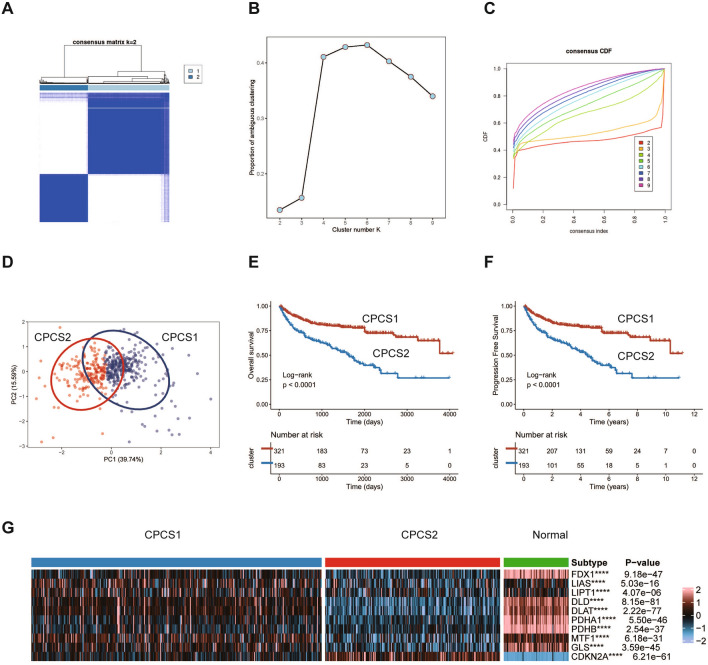
Establishment of two clusters for copper-induced cell death-related genes in ccRCC. **A** Consensus matrix of samples in TCGA-ccRCC for k = 2. **B** The cluster numbers are determined by the lowest proportion of ambiguous clustering. **C** The cumulative distribution function curves, k = 2 to 9. **D** The principal component plot is based on copper-induced cell death-related genes. **E**, **F** Survival analysis for overall survival (left) and progression-free survival of the two subtypes in the TCGA-ccRCC dataset. **G** The expression profiles of copper-induced cell death-related genes in two subtypes and normal samples

As we found above, copper-induced cell death-related genes were protective factors for ccRCC, which was unique and different from other cancers. Thus, we investigated the characteristics of copper-induced cell death-related genes in ccRCC. The TCGA ccRCC samples were classified into several subtypes using an unsupervised clustering method based on the expression levels of copper-induced cell death-related genes. The optimal classification method was validated, and the PAC method was used to assess the robustness of the analysis. Consequently, the TCGA ccRCC dataset was divided into two subtypes, namely, copper-pattern cancer type 1 (CPCS1) and type 2 (CPCS2) (Fig. 3A–D). After excluding patients lacking tumor stage and grade information, the clinicopathological characteristics of the 506 ccRCC patients with the two subtypes were compared, as shown in Additional file 2: Table S2. Compared with CPCS1 subtype patients, CPCS2 subtype patients had higher T stage and shorter overall survival (OS) and progression-free survival (PFS) (Fig. 3E, F). We analysed the expression of copper-induced cell death-related genes in ccRCC subtypes and normal kidney tissues. The CSP2 subtype, similar to the desert of copper, induced cell death and expressed lower levels of copper-induced cell death-related genes than the CPCS1 subtype and normal tissues. Conversely, CDKN2A showed higher expression levels in the CPCS2 subtype than in CPCS1. The desert of copper-induced cell death-related genes in CPCS2 contributed to the suppression of copper-induced death, which trained CPCS2 to aggressive clinical subtypes.

### Functional enrichment analysis of ccRCC subtypes

**Fig. 4:**
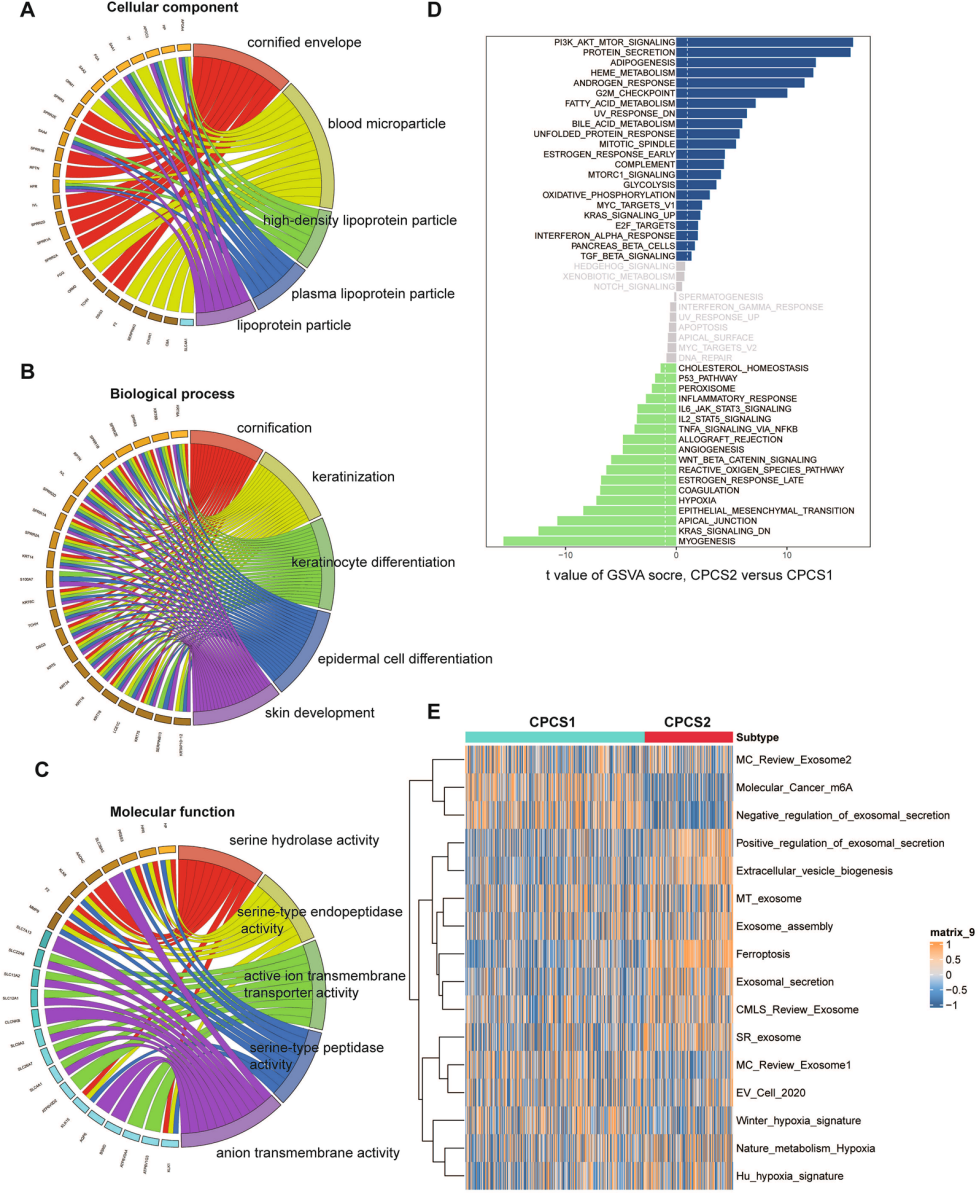
Functional enrichment analysis of ccRCC subtypes. GO enrichment analysis between the two subtypes in cellular component (**A**), biological process (**B**) and molecular function **C**. **D** GSEA pathway analysis between the two subtypes. **E** Tumor microenvironment-related pathways

Since there were differences in the copper-induced cell death profiles and clinical characteristics between the subgroups, we next investigated the biological function and hallmarks of CPCS1 and CPCS2. GO analysis demonstrated that differentially expressed genes were mainly enriched in cornified envelope, blood microparticle and lipoprotein particle in cellular component; cornification, keratinization, and epidermal cell differentiation in biological process; and serine hydrolase activity, active ion transmembrane transporter activity and anion transmembrane activity in molecular function (Fig. 4A–C). GSEA pathway analysis indicated that these genes were mainly enriched in cellular responses to external stimuli, metabolism of lipids and vesicle-mediated transport pathways (Additional file 1: Fig. S4A). Compared with the CPCS1 subtype, the CPCS2 subtype was more correlated with the PI3K-AKT-mTOR, fatty acid metabolism, oxidative phosphorylation and KRAS and MYC pathways, while the CPCS1 subtype was more related to the myogenesis, EMT, hypoxia and P53 pathways (Fig. 4D). The CSP2 subtype, but not the CPCS1 subtype, was activated in tumorigenicity and cancer progression.

The tumor microenvironment (TME) and metabolic pathways were compared between the CPCS1 and CPCS2 subtypes. The CSP2 subtype was significantly activated in exosome secretion, ferroptosis and extracellular vesicle biogenesis and was inhibited in m6A methylation modification (Fig. 4E). The TME fraction pathways included EMT, CAF, and cytokine signaling pathways. The CSP2 subtype was stimulated for immune checkpoint, CD8 T cells, chemokines, and interleukin receptors. The CSCP1 subtype was associated with mast cells, EMT, WNT and TGF-β receptors (Figure S4B). Metabolic processes play crucial roles in ccRCC [33]. We observed activation of multiple metabolic pathways in CPCS2 subtypes, including amino sugar and nucleotide sugar metabolism, sulfur metabolism, oxidative phosphorylation, glutathione metabolism, cardiolipin metabolism, and valine, leucine and riboflavin metabolism. The CPCS1 subtype was stimulated for inositol phosphate metabolism, lysine degradation, remethylation and terpenoid backbone biosynthesis. The TME and metabolic environment shape the different ccRCC subtypes to a certain degree.

### Comparison of immune infiltration characteristics between subtypes

**Fig. 5:**
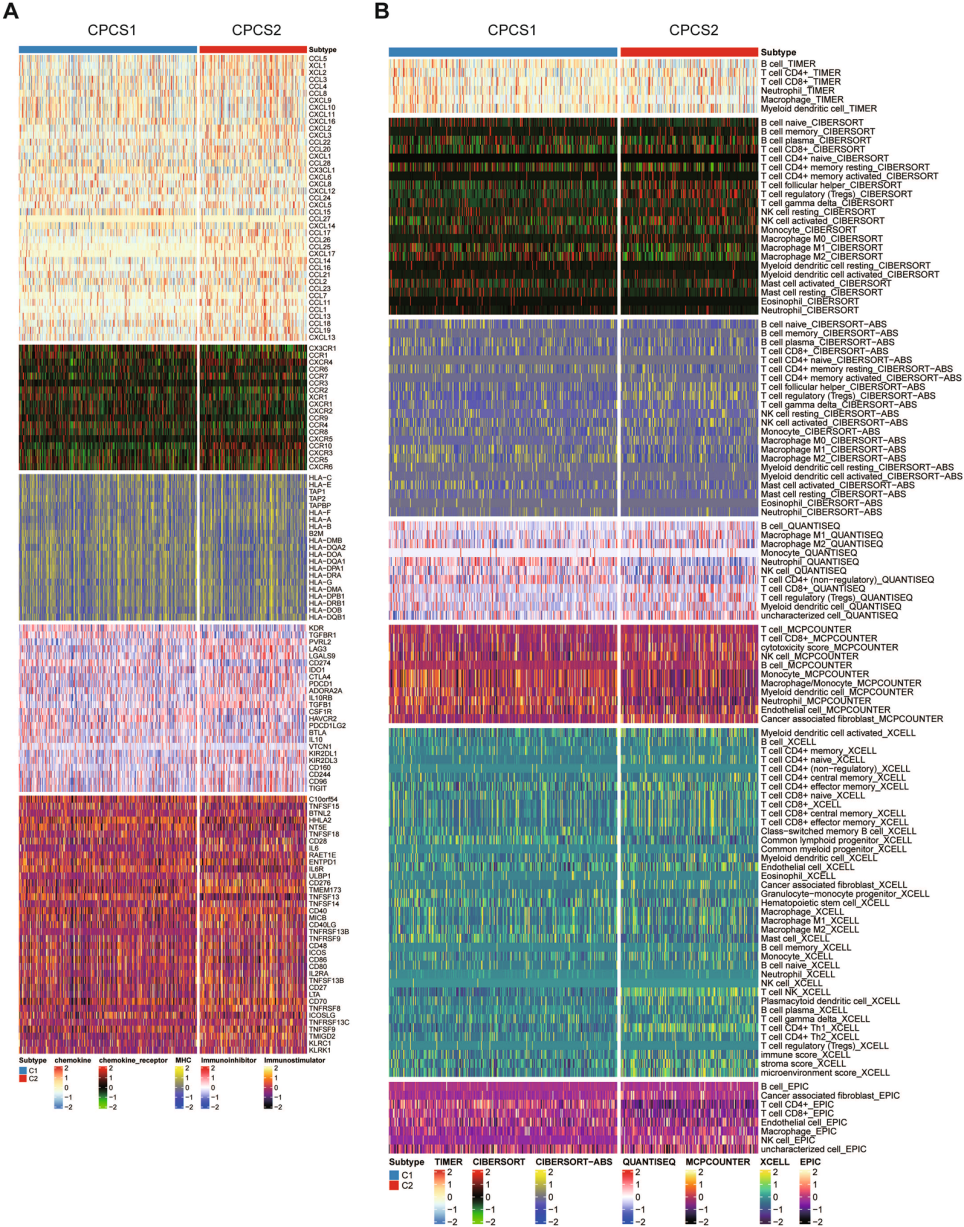
Investigations of immune profiling. **A** Heatmap of immune-related genes between CPCS1 and CPCS2. **B** Heatmap of tumor-related infiltrating immune cells based on the TIMER, CIBERSORT, CIBERSORT-ABS, QUANTISEQ, MCPCOUNTER, XCELL, and EPIC algorithms in the two subtypes

**Fig. 6:**
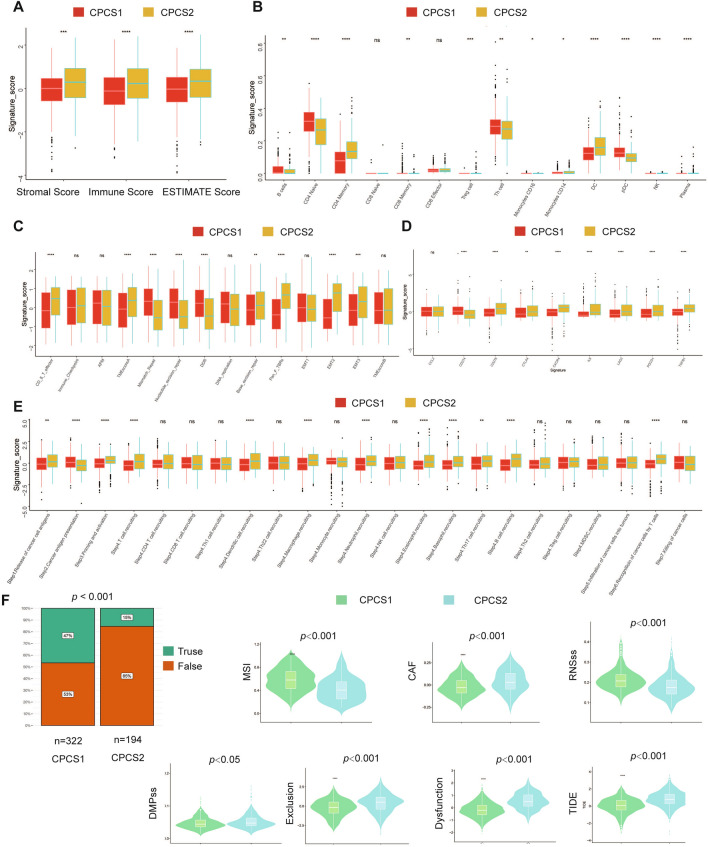
Landscapes of specific immune components and immune function scores. **A** ESTIMATE scores of the two subtypes. **B**–**E** The immune cells, immune pathways, immune antigens, and tumor kill steps between the two subtypes. **F** The immune function scores between the two subtypes

Immunotherapy has gradually become the main characteristic of ccRCC treatment in recent years. To define the effect of copper-induced death on immune profiling, we analysed the immune infiltration environment of the two subgroups using GSVA. Immune-related genes of the CPCS2 subtype showed an overall upwards-regulated trend compared with the CPCS1 subtype. The CSP2 subtype expressed higher levels of CCL5, CCL21, CCL26, CXCR4, CXCR5, CXCR10, IL10RB, LAG3, CD276, CD48, CD70 and TCFRSF8 (Fig. 5A). Similar results were seen in several immune infiltration scoring models, including TIMER and CIBERSORT, QUANTISEQ, XCELL and EPIC. The CSP2 subtype was correlated with CD8 + T cells, Tregs, cancer-associated fibroblasts and NK cells (Fig. 5B). The above investigation confirmed the greater immune infiltration in CPCS2 than in CPCS1. Consistently, ESTIMATE algorithm analysis showed that the CPCS2 subtype contained a higher stromal score, immune score, and ESTIMATE score (Fig. 6A). The specific immune components were compared between CPCS1 and CPCS2 subtypes. The CSP2 subtype lacked activated dendritic cells, which prompted the reduction of identifying tumor cells (Fig. 6B). The CSP2 subtype was less capable of repairing DNA damage (Fig. 6C). The CSPC2 subtype expressed more CD276, IL-6, PDCD1 and TGFB1, while CD274 (PD-L1) expression was significantly downregulated (Fig. 6D). In the antitumour processes, the CPCS2 subtype expressed a higher degree of immune infiltration, but tumor clearance abilities were severely impaired, possibly due to the immunosuppressive states (Fig. 6E). The immune function scores were also evaluated in the two subtypes. The CSP1 subtype gained a higher microsatellite instability (MSI) score, whereas the CPCS2 subtype obtained a higher cancer-associated fibroblast (CAF) score, stemness-associated score, dysfunction score and tumor immune dysfunction and rejection score (TIDE) (Fig. 6F). Nearly half of the cases in CPCS1, a copper-induced death-activated subtype, developed an effective immune response. Conceivably, the C2 subtype may generate more effective immune responses by activating copper-induced death to form abundant presented antigens.

### Comparison of tumor somatic mutations and CNVs in two subtypes

**Fig. 7:**
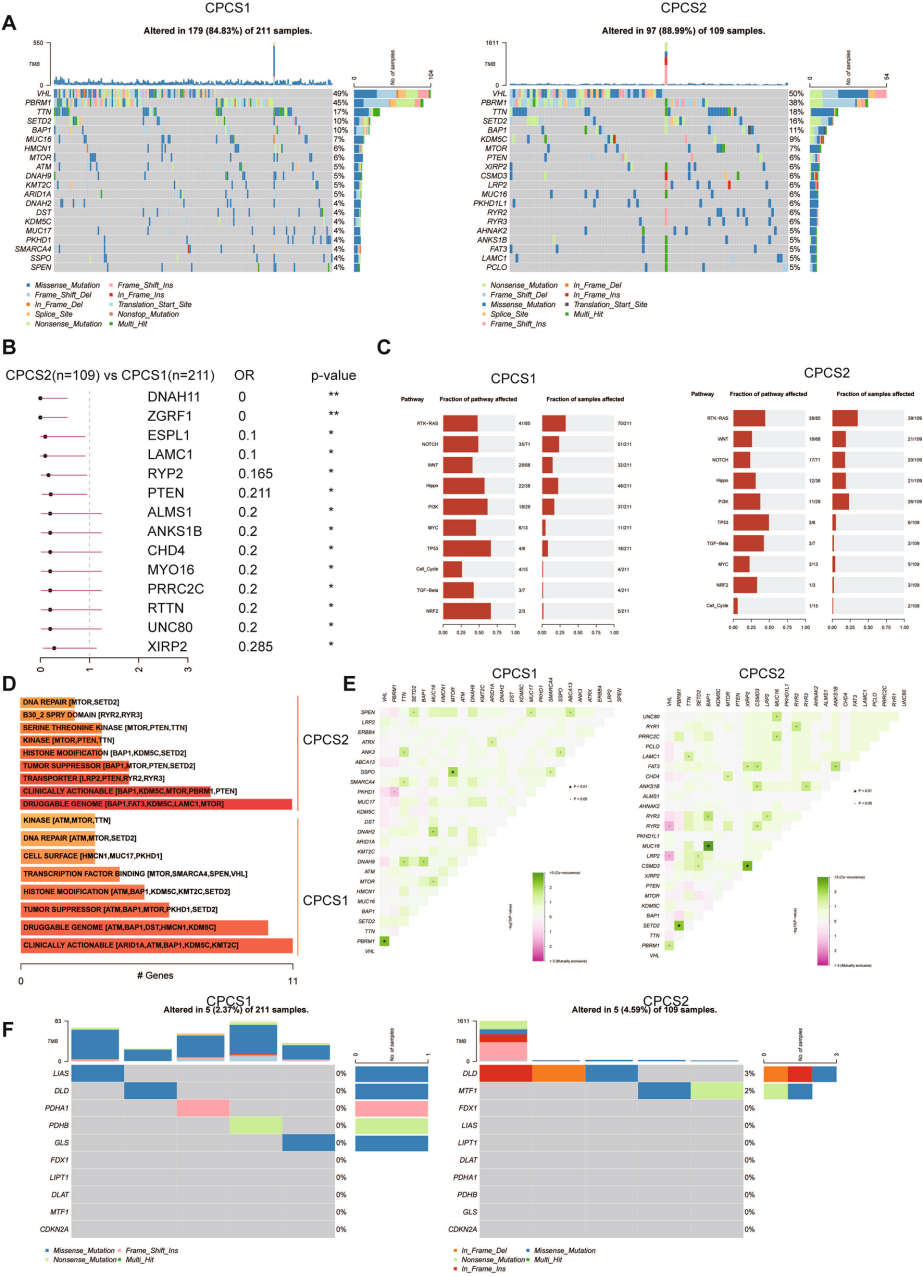
Landscapes of somatic mutations and potential targets in the two subtypes. **A** Waterfall plot showing the mutation patterns of the top 20 most frequently mutated genes. **B** Forest analysis indicating differentially mutated genes between the two subtypes. **C** The fraction of pathways or samples of oncogenic signaling pathways in CPCS1 and CPCS2. **D** Potential druggable gene categories from the mutation dataset in CPCS1 and CPCS2. **E** The synthetic lethal mutations in CPCS1 and CPCS2. **F** Waterfall plot showing the mutation patterns of copper-induced cell death-related genes in CPCS1 and CPCS2

In addition to the influence of the microenvironment on drug therapy, genome mutations are also core factors in drug effectiveness. We examined the differential distribution of tumor somatic mutations between the two subtypes. The gene mutations of the CPCS2 subtype were more frequent than those of the CPCS1 subtype (Fig. 7A). The picture depicts the mutation frequency of the top 20 mutant genes. VHL, PBRM1, TTN, SETD2 and BAP1 were the most frequently mutated genes for both subtypes. Compared to CPCS1, the CPCS2 subtype had several genes with a higher mutation frequency, including KDM5C (9% vs 4%), PTEN (6% vs ≤ 4%), XIRP (6% vs ≤ 4%), LPR2 (6% vs ≤ 4%), RYR2/3 (6% vs ≤ 4%) and ANKS1B (5% vs ≤ 4%). The forest analysis also confirmed the above findings. As shown in Fig. 7B, the CPCS2 subtype was more mutated in DNAH11, ZGRF1, ESPL1, LAMC1, RYP2, PTEN and ANKS1B. The fraction of pathways affected in CPCS1 was more frequent than that in CPCS2, while CPCS2 shared a greater fraction of affected samples (Fig. 7C). For instance, the CPCS1 subtype was more frequent than the CPCS2 subtype for the fraction of pathways affected in NOTCH (35/71 vs 17/71), NOTCH (28/68 vs 18/68), Hippo (22/38 vs 12/38) and MYC (6/13 vs 3/13). The CPCS2 subtype was more frequent for the fraction of samples affected in WNT (21/109 vs 51/211). The DGldb database was used to investigate potential therapeutic targets of mutated genes. The potential therapeutic targets of the CPCS1 subtype included ARID1A, ATM, BAP1, KDM5C and KMT2C, while CPCS2 targets were mainly BAP1, FAT3, KDM5C, LAMPCCS1 and mTOR (Fig. 7D). The somatic interactions analysis suggested that comutation of PBRM1 and PKHD1 caused cell death in CPCS1, and comutation of VHL and RYR2/LRP2 also led to death (Fig. 7E). These synthetic lethal mutations could potentially be used to develop treatments for different subtypes. For mutations of copper-induced cell death-related genes, the CPCS2 subtype reserved a higher mutation frequency (4.59% vs 2.37%) and more abundant mutation types than the CPCS1 subtype (Fig. 7F). The DLD gene contained frame deletion, frame insertion and missense mutations in the CPCS2 subtype.

**Fig. 8:**
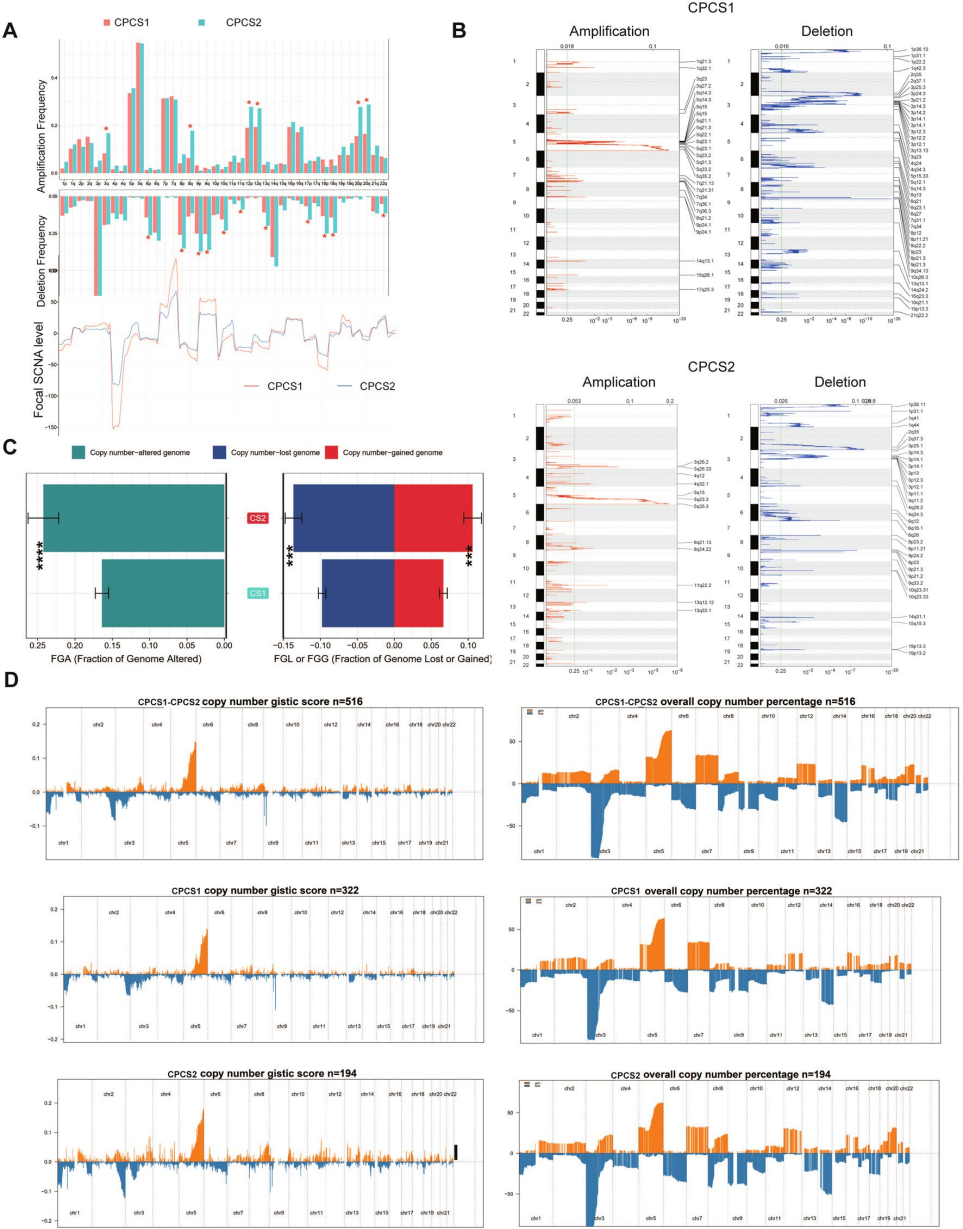
Landscapes of copy number variations in the two subtypes. (**A**) The amplification or deletion frequency in chromosomes between the two subtypes. **B** Specific amplification or deletion location in CPCS1 and CPCS2. **C** Bar plot of genomic fractions altered in the two subtypes. **D** The GISTIC score and percentage of copy number profiles in ccRCC

Copy number variations and not just gene somatic mutations were also compared between the two subtypes. For amplification frequencies, the CPCS2 subtype presented higher CNV frequencies than the CPCS1 subtype on chromosomes 3q, 8q, 12p, 12q, 20p, and 20q. Regarding deletion frequencies, the CPCS2 subtype contained significant CNV frequencies on chromosomes 6p, 8p, 9p, 9q, 10q, 11q, 13q, 17p, 18p, 18q, and 22q (Fig. 8A). The amplification and deletion regions on chromosomes were decoded and analysed using GISTIC 2.0 software (Fig. 8B–D, Additional file 3: Table S3). The recurrent CNVs of CPCS1 included amplification of 5q35.2 (CPEB4), 5q31.3 (KCTD16), 5q33.2 (SGCD), 5q15 (NR2F1), 14q13.1 (CFL2, NFKBIA, PSMA6, SRP54, PPP2R3C), and 7q34 (EPHB6, TRPV6) and deletion of 9p21.3 (CDKN2A), 1p36.13 (UBR4), 9p21.3 (CDKN2B), 9p23 (PTPRD), 2q37.1 (ALPI, COL4A3, GPR35, PTPRN), and 3p21.2 (ABHD14A, PARP3, RBM15B). The specific CNVs of CPCS2 were the amplification of 5q35.3 (FGFR4, HNRNPH1, MAPK9, RNF44), 5q23.3 (HINT1), 11q22.2 (MMP7), 3q26.33 (PIK3CA, ZNF639), and 8q24.22 (ADCY8, ADCY8, GPR20) and the deletion of 2q37.3 (AGXT, KIF1A), 9p23 (PTPRD), 1p31.1 (NEGR1), 1p36.11 (C1QA, CD52, GPR3, RUNX3), and 9p21.3 (CDKN2A, CDKN2B) (Fig. 8B–D). Differences in the amplification and deletion of genomic regions may lead to the formation of the two subtypes.

### Drug sensitivity analysis of two subtypes

**Fig. 9:**
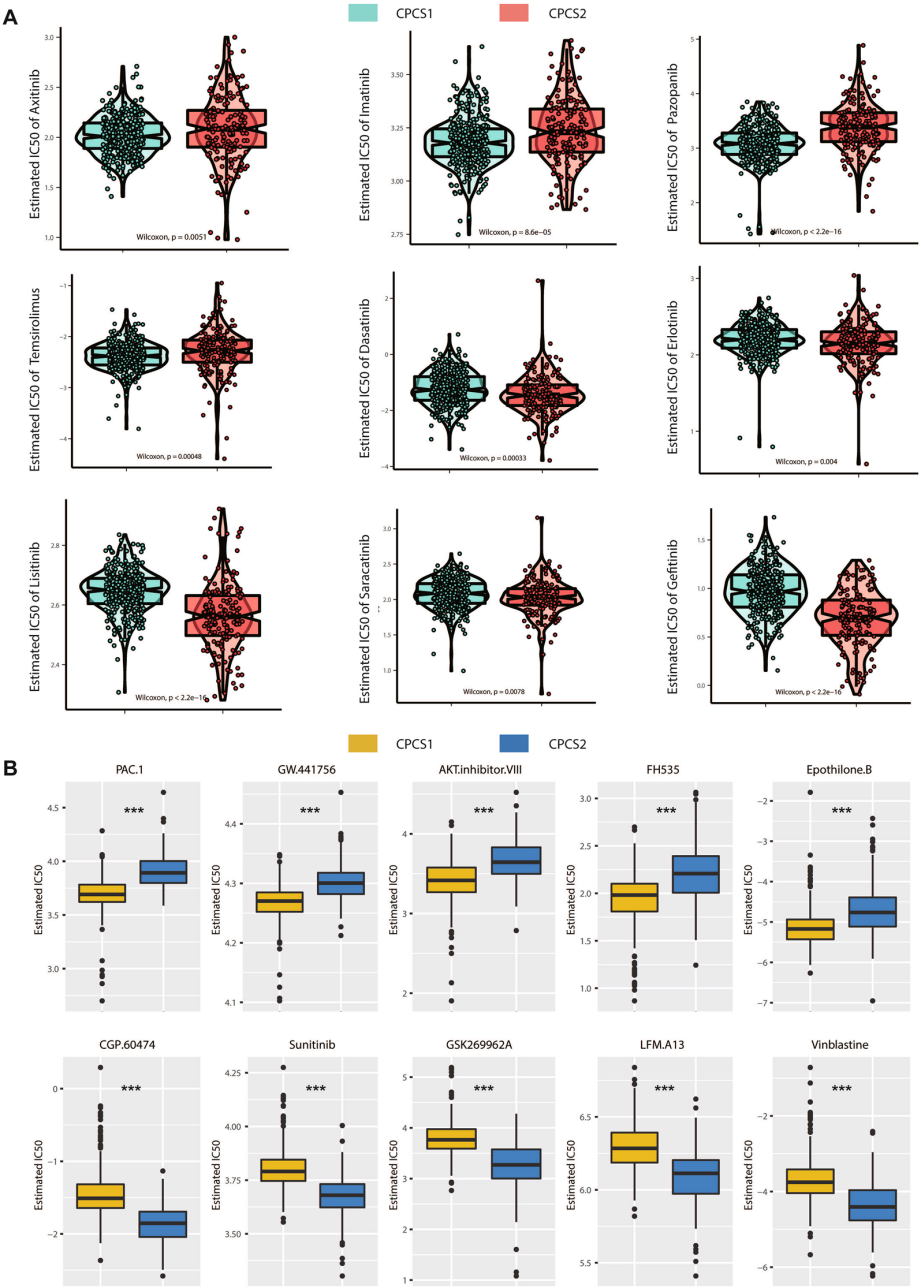
Drug sensitivity analysis of the two subtypes. **A** Estimated IC50 of the indicated molecular targeted drugs in CPCS1 and CPCS2. **B** Estimated IC50 of the potential drugs in CPCS1 and CPCS2

After comprehensively inspecting the clinical characteristics, prognosis, immune profile and mutation information of the two subtypes, we further searched for sensitive targets and potential drugs of the two subtypes based on the above investigations. The GDSC database was used to perform drug sensitivity analysis of the two ccRCC subtypes. Significantly different responses were observed between the CPCS1 and CPCS2 subtypes. The CSP1 subtype was more sensitive to axitinib, crizotinib, pazopanib and temsirolimus, while the CPCS2 subtype displayed sensitivity to dasatinib, erlotinib, lisitinib, saracatinib and gefitinib (Fig. 9A). Although most of them were TKI inhibitors, there were certain differences in the effective targets of CPCS1 and CPCS2 subtypes, which were VEGFR/PDGFR/MET and EGFR/SRC, respectively. We further analysed two subtypes of potential molecular inhibitor drugs. CPCS1 subtype indicated sensitivity to PAC.1, GW.441756, AKT inhibitor VIII, FH535 and Epothione. B, while the CPCS2 subtype was more responsive to CGP.6047, sunitinib, GSK269962A, and LFM. A13 and vinblastine (Fig. 9B). We then investigated potential drugs targeting the oncogenic process. The CellMiner database was utilized to determine the relationships between copper-induced death-related genes and drug sensitivities. Negative correlations were observed between GLS expression and the IC50 of TYROTHRICIN, EMD-534085, Batasertib and PDHA1 expression and the IC50 of LY-3023414 (Additional file 1: Fig. S5A). The results suggested that these were appropriate for ccRCC patients with high expression of GLS and PDHA1. In addition, MI-503, LY-3154567 or KPT-9274 may be suitable for patents with low DLD, CDKN2A or DLAT expression, respectively.

### Verification of classification model in external dataset

**Fig. 10:**
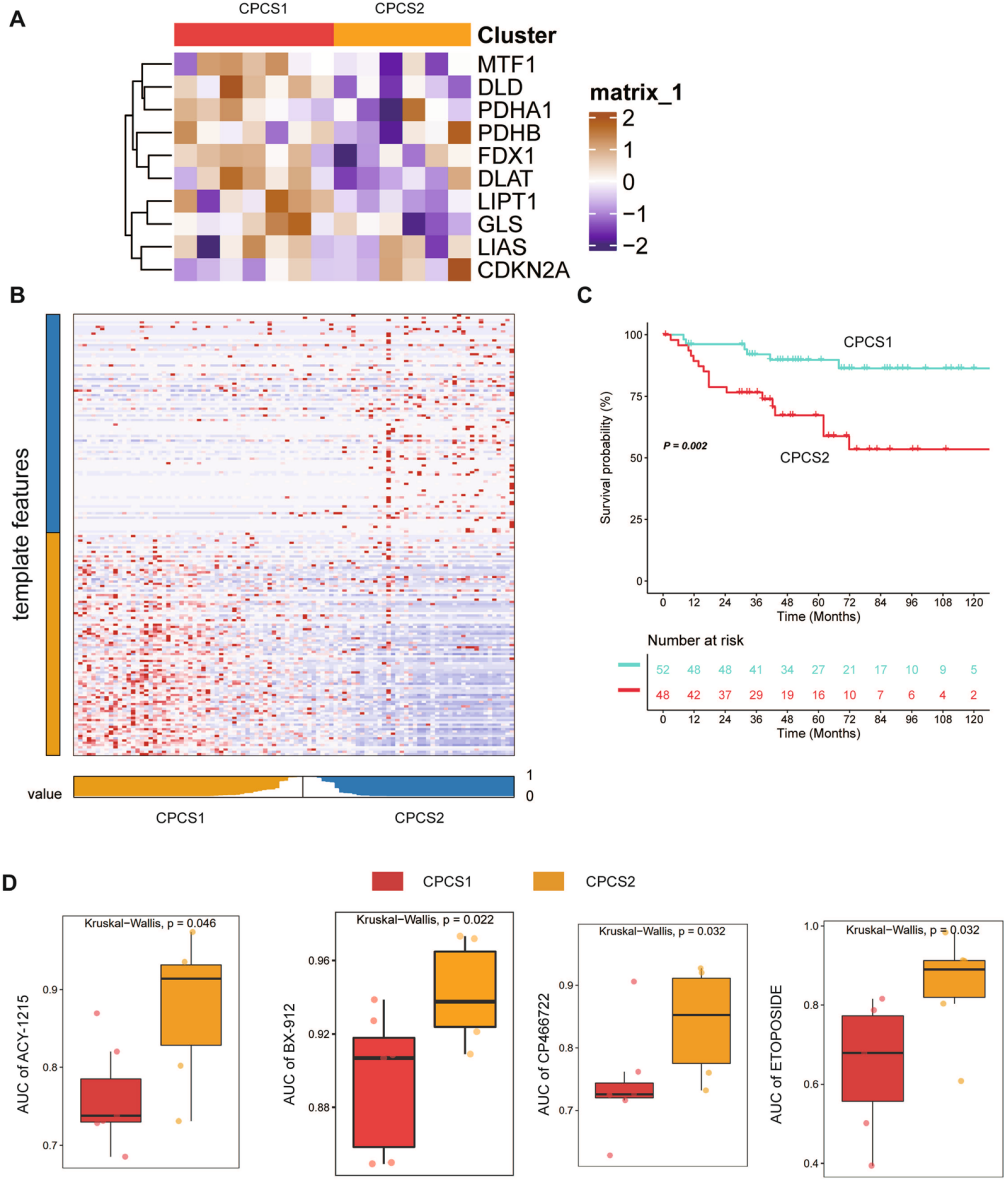
Verification of the classification model in the external dataset. **A** Heatmap of the expression profiles of copper-induced death-related genes in the two subtypes of GDSC renal cancer cells. **B** Heatmap of NTP in the JAPAN-KIRC cohort using subtype-specific upregulated hallmarks identified from the TCGA-ccRCC cohort. **C** Survival analysis of the two predicted subtypes of ccRCC in the JAPAN-KIRC cohort. **D** Drug sensitivity values in the form of normalized AUC using the GDSC renal cancer cell database

To further confirm the robustness of the classification model, we performed identification using the GDSC ccRCC cell line database and JAPAN-KIRC cohort. A significant difference was observed in cell lines between CPCS1 and CPCS2 subtypes. Most copper-induced cell death-related genes except CDKN2A were downregulated in CPCS2, similar to the TCGA cohort (Fig. 10A). The nearest template prediction (NTP) algorithm suggested that the dysregulated hallmarks identified from ccRCC subtyping can divide the JAPAN-KIRC cohort into two different groups (Fig. 10B). The CSP2 subtype predicted a poorer prognosis of renal cancer patients than the CPCS1 subtype, which was consistent with previous data (Fig. 10C). Compared to CPCS2, the CPCS1 subtype displayed more sensitivity to most tested drugs (ACY-1215, BX-912, CP466722, ETOPOSIDE, TEMSIROLIMUS, LESTAURTINIB, QS11, KIN001-206, PALBOCICLIB, and SN-38) (Fig. 10D, Additional file 1: Fig. S5B, Additional file 4: Table S4). The results suggested the effectiveness of targeting the CPCS1 subtype.

### Construction of a four-copper-induced-cell death-related genes risk model

**Fig. 11:**
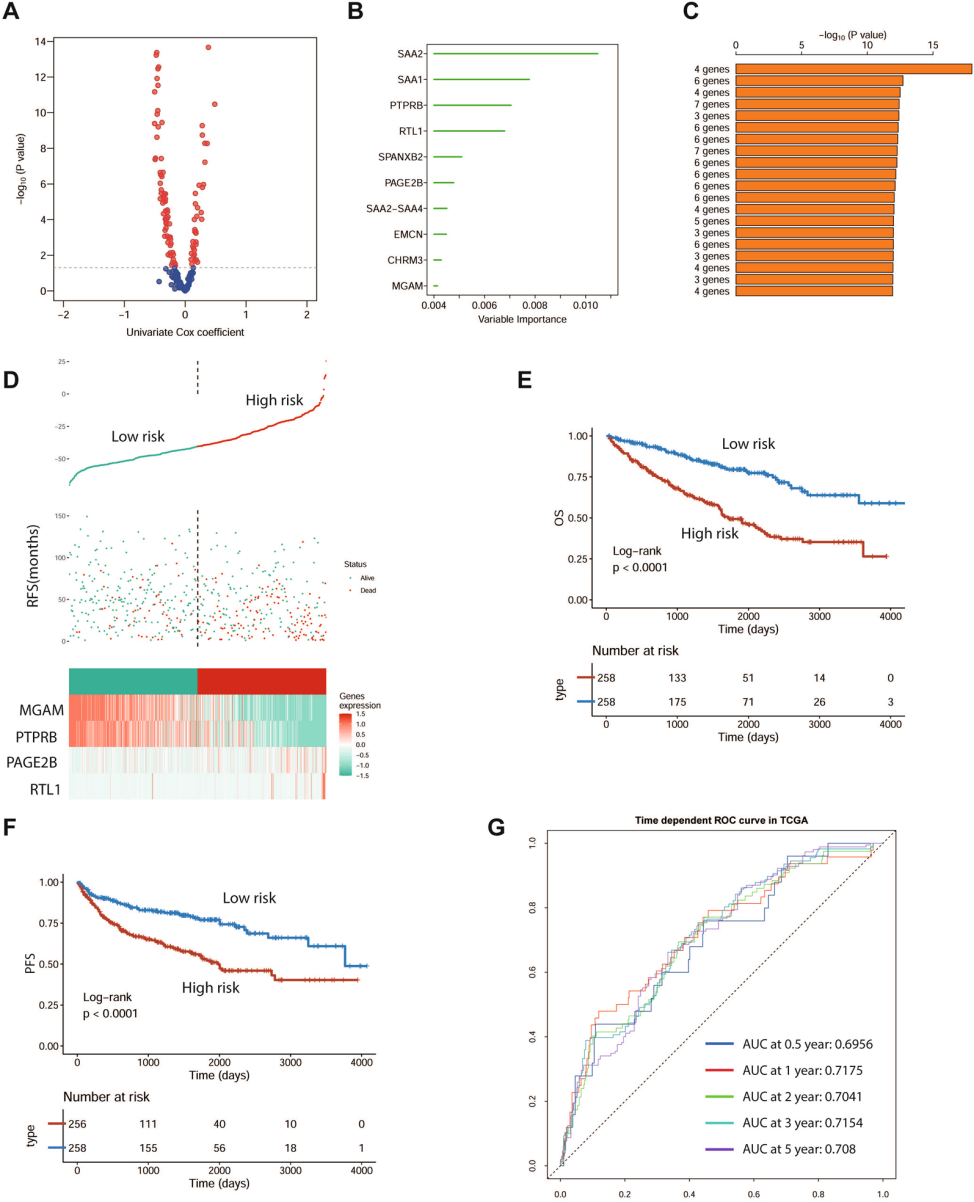
Construction of a four-copper-induced cell death-related gene risk model. **A** Volcano plot showing the dysregulated biomarkers between the two subtypes by univariable Cox regression analysis. **B** Random survival forest analysis screening 10 genes. **C** Based on various combination analyses, the top 20 signatures are ordered by the p value. **D** Risk score analysis in the TCGA-ccRCC cohort. **E**–**F** Survival analysis for OS **E** and PFS **F** of the two risk signatures in the TCGA-ccRCC cohort. **G** The time-dependent ROC curves for the two risk signatures in the TCGA-ccRCC cohort

To further evaluate the reliability of the subtyping model, univariate Cox regression analysis was used to explore dysregulated biomarkers between the two subtype samples (Fig. 11A). The random forest supervised classification algorithm identified the 10 most relevant genes (Fig. 11B). To establish the best risk assessment model, we performed Kaplan‒Meier (KM) analysis, and based on the p value of every model, we finally screened out a risk assessment model composed of four genes (MGAM, PTPRB, PAGE2B, RTL1), named RCC-CUPT4 (Fig. 11C, D). The risk score of each patient was calculated as follows: RCC-CUPT4 = − 7.553304*MGAM-6.184020*PTPRB + 3.895654*PAGE2B + 4.645926*RTL1. To further identify the accuracy of RCC-CUPT4, both TCGA-ccRCC and JAPAN-KIRC cohorts were divided into high-risk and low-risk groups according to median scores (Additional file 1: Fig. S9A). The high-risk group predicted worse OS and PFS than the low-risk group in both cohorts (Fig. 11E, F, Additional file 1: Fig. S6B). The area under the ROC curves also confirmed the high sensitivity and specificity of the RCC-CUPT4 model for predicting prognosis. The AUC scores for the TCGA ccRCC cohort were 0.6956, 0.7175, 0.7041, 0.7154 and 0.705 at 0.5 years, 1 year, 2 years, 3 years and 5 years, respectively (Fig. 11G). Better AUCs were obtained in the JAPAN-KIRC cohort, and the AUC scores were 0.9485, 0.733, 0.8478, 0.7804 and 0.7485 at 0.5 years, 1 year, 2 years, 3 years and 5 years, respectively (Additional file 1: Fig. S6C). The above results confirmed the reliability and practicality of our classification.

### The core role of DLAT and functional verification

**Fig. 12:**
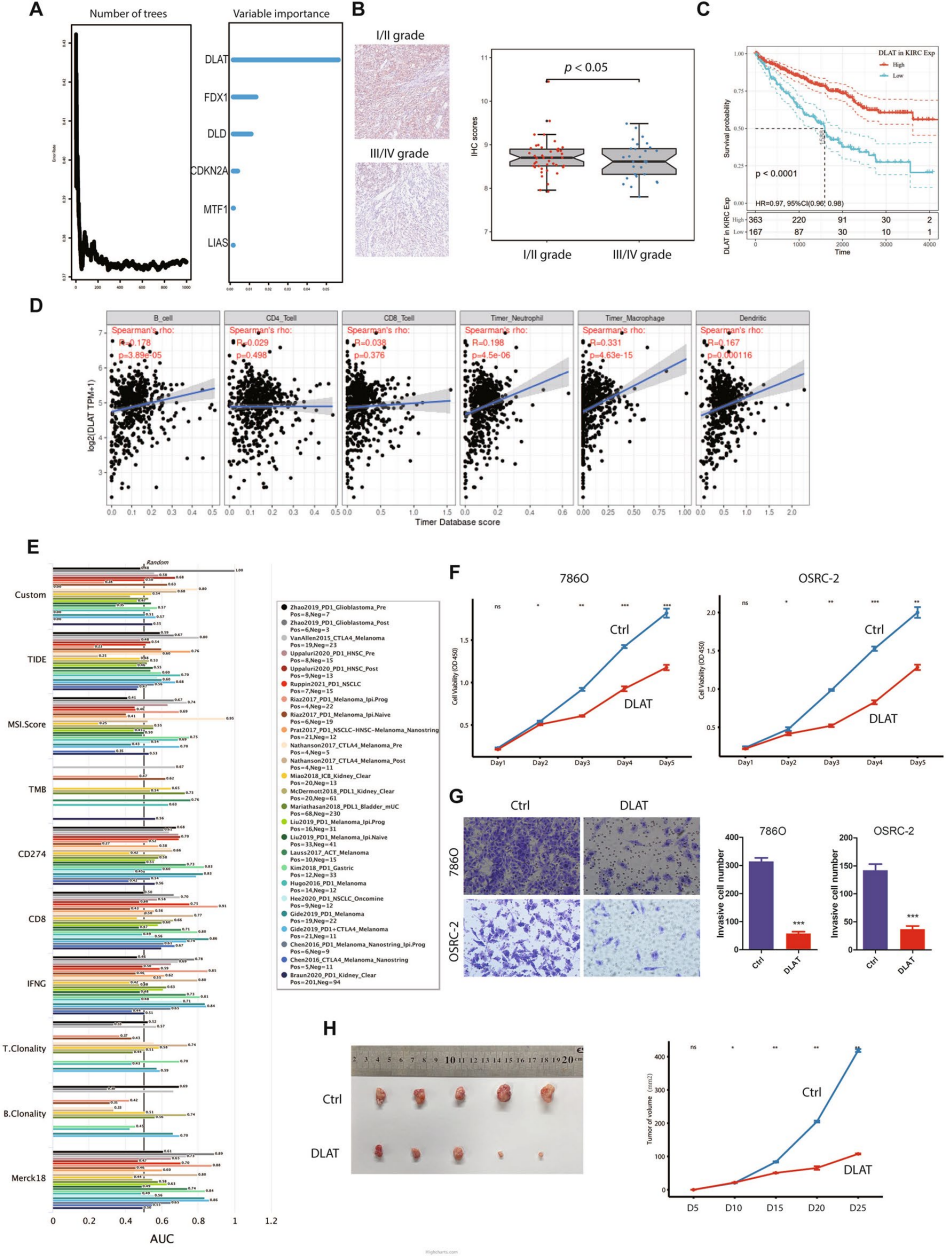
The core role of DLAT and functional verification. **A**) Number of trees indicating the importance proportion of copper-induced cell death-related genes. B Immunohistochemical score of DLAT in high-stage and low-stage ccRCC. **C** Survival analysis for OS of DLAT expression. **D** The association between DLAT expression and immune cell infiltration in ccRCC. **E** The comparison among several classic cancer risk prediction models in the form of AUC scores. (**F**, **G**) Cell proliferation (**F**) and invasion (**G**) of 786° and OSRC-2 cells after transfection with NC and overexpressed DLAT lentivirus. **H** Subcutaneous xenograft models were established, and the tumor weight and growth curve of 786° cells infected with NC and overexpressed DLAT lentivirus

In view of the importance of copper-induced cell death in ccRCC, we evaluated which gene shared the most importance proportion in clinical outcome. Unlike previous studies, DLAT rather than FDX1 may play the core role among copper death signatures when we performed random forest analysis (Fig. 12A); a similar result was shown in the JAPAN-KIRC cohort (Additional file 1: Fig. S7A). As previously observed, DLAT was downregulated in renal cancer, and the magnitude of regulation increased with the stage (Additional file 1: Fig. S7B, C). The immunohistochemical score of DLAT was downregulated in high-stage ccRCC compared with low-stage ccRCC (p < 0.05) (Fig. 12B, Additional file 1: Fig. S7D). Accordingly, the high expression of DLAT predicted good prognosis of ccRCC and was a protective factor of ccRCC (Fig. 12C). The TIMER database found that DLAT was strongly correlated with neutrophils, macrophages, and DCs (Fig. 12D). In addition, we evaluated the AUC efficacy of DLAT with several classic cancer risk prediction models and the effect of DLAT mutation on immune cell infiltration in ccRCC (Figs. 12E, Additional file 1: Fig. S7F). The AUC curve of DLAT was 0.54 in Miao Kidney (ICB) data, while the scores achieved 1.00 in Zhao glioblastoma (PD-1) and 0.80 in Nathanson melanoma (CTLA4). In addition, DLAT expression may significantly influence multiple immune signatures across cancers, especially in ccRCC (Figure S7G). To verify the function of DLAT in renal cancer cell lines, we constructed a DLAT-overexpressing lentivirus (DLAT). Using the CCK-8 assay, overexpression of DLAT significantly inhibited the proliferation of ccRCC cell lines (p < 0.001) (Fig. 12F). Transwell assays showed that overexpression of DLAT significantly reduced the migration ability of ccRCC cell lines (p < 0.001) (Fig. 12G). In vivo experiments found that upregulation of DLAT inhibited the increase in the volume and weight of xenografts in mice (p < 0.01) (Fig. 12H). These results suggested that upregulation of DLAT expression could effectively inhibit the growth and metastasis of renal cancer. Therefore, it makes sense to prompt copper-induced death by activating DLAT expression to achieve the goal of tumor eradication.

### Cuprotosis in ccRCC could enhance tumor immunity though cGAS-STING signaling

**Fig. 13:**
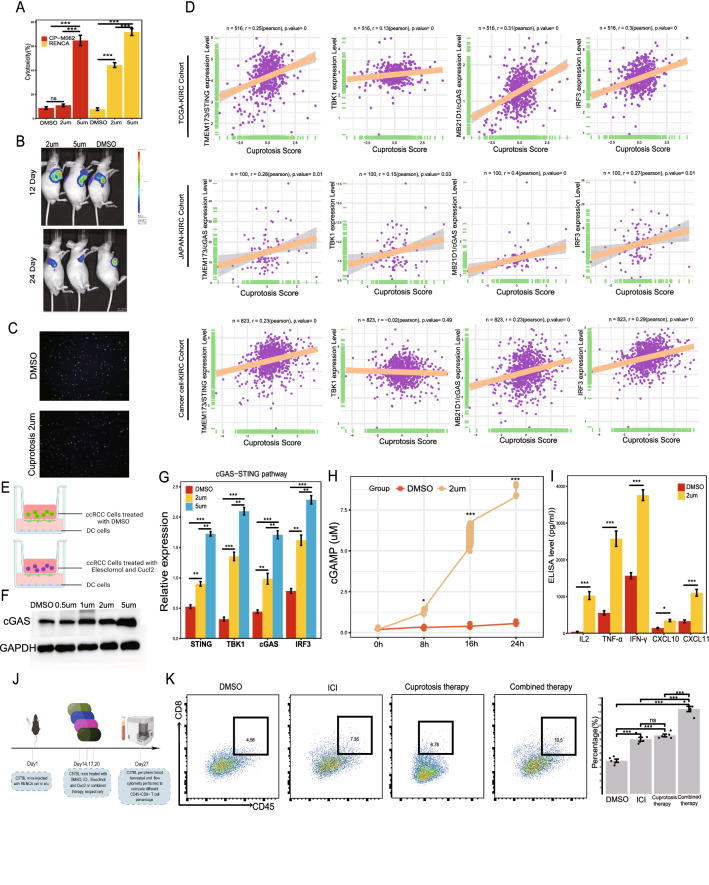
Activation of cuprotosis enhances ccRCC tumor immunity. **A** The Cell cytotoxicity was measured by lactate dehydrogenase (LDH) release assay after DMSO, elesclomol and Cucl2 for 48 h between CP-M062 and RENCA, to identify optimal concentration of cuprotosis inducer agent. **B** Tumor-bearing mice were treated with DMSO or elesclomol and Cucl2, and the total fluorescence intensity of each mouse model was recorded. **C** Comparison of the proportion of EdU positive cells in RENCA treated with DMSO or elesclomol and Cucl2. **D** Correlation between the cuprotosis score and cGAS-STING signature related genes expression level. **E** Schematic diagram of the coculture system of tumor and DC cells. **F** Different cGAS expression levels of DCs cells after cocultured with tumor cells pre-treated with different concentration level of elesclomol and Cucl2. **G** Expression levels of cGAS-STING signatures of DCs harvested from co-culture system were measured by qRT‑PCR. **H** Different cGAMP level from medium supernatant of co-culture system with tumor cell pre-treated with DMSO or elesclomol and Cucl2. **I** The IL-2, TNF-α, IFN-γ, CXCL10 and CXCL11 protein levels in the coculture medium supernatant were measured by ELISA after 48 h of coculture. **J** Schematic diagram of flow cytometry. **K** Representative flow cytometry plots of the percentage of CD45^+^CD8^+^ T cells from peripheral blood from different mice groups treated with DMSO, anti-PD-1mAB, cuprotosis inducer reagents, and combined therapy (anti-PD-1 + cuprotosis inducer reagents) by intraperitoneal, respectively. 2 μM, 5 μM: elesclomol and Cucl2 at 2 and 5 μmol/L. *P < 0.05, **P < 0.01, ***P < 0.001, ns: no significance

The results mentioned above reminded us that CPCS1, an activated cuproptosis phenotype of ccRCC, led to a better prognosis, enhanced antitumor immunity and a higher response rate to ICI therapy. Cuproptosis functioned as a novel programmed cell type. Figure 6C also indicates that the DNA damage repair signal was activated in CPCS1; we thus speculated that cuproptosis could activate tumor immunity through DDR-related signatures, which then stimulated tumor immunity through cGAS-STING signaling. Cell cytotoxicity was performed to choose the effective and safe concentration of cuproptosis inducer agent, and we applied 2 μmol/L as the optimal concentration for the activation of cuproptosis for RENCA, since the cytotoxicity rate of RENCA was higher than CP-M062 at 2 μmol/L (Fig. 13A). Activated cuproptosis significantly inhibited tumor growth in vitro and vivo (Fig. 13B, C, Additional file 1: Fig. S8A, B). Through three independent ccRCC datasets, (TCGA-KIRC, JAPAN-KIRC and Cacner cell-cohort), containing 1439 ccRCC samples, we found that the cuproptosis score calculated by ssGSEA was significantly correlated with the cGAS-STING-related signature, including TMEM173/STING, TBK1, MB21D1/cGAS and IRF3, except for TBK1 in Motzer’s cohort (Fig. 13D). Similar to the results in Fig. 2C, the cuproptosis score was lower in malignant cells in ccRCC and higher in tumor and stromal cells at single cell level (Figure S8C, D). Previous studies have indicated that cGAS-STING signaling was correlated with DC in anti-tumor immunity [34–38]. We thus adopted the three ccRCC cohorts to investigate correlation between cuproptosis score and DC signatures, which also showed that cuproptosis score was significantly positive correlated with DC infiltration score (Figure S9A).

We next applied a co-culture system of DC and renal cancer cells to verify our findings (Fig. 13E). Expression of the cGAS-STING pathway at both the protein and RNA levels was increased in a dose-dependent pattern in DCs cocultured with cuproptosis-activated tumor cells (Fig. 13F, G). In addition, the intracellular activity level of cGAMP in DCs was higher in the cuprotosis-treated group (Fig. 13H). The secretion levels of IL2, TNF-α, IFN-γ, CXCL10 and CXCL11 were increased in medium supernatant from co-culture system (Fig. 13I). 547-553As Fig. 13J indicated that, at 14 days after RENCA cells inoculation in C57BL/6 mice, all mice carrying similar size of tumors were randomized into four groups receiving DMSO, anti-PD-1mAB, cuprotosis inducer reagents, and combined therapy (anti-PD-1 and cuprotosis inducer reagents) by intraperitoneal (i.p., given at days 14, 17, 20), respectively. After one week, all mice treated with those agents were euthanized and peripheral blood were harvested to quantity the percentage of CD8 + T cells among different groups. Finally, injection of cuproptosis induce pre-treated RENCA could increase the percentage of CD45^+^CD8^+^ T cells from peripheral blood, and combined therapy with cuproptosis and ICI further increased this percentage (Fig. 13K). All these results suggested that cuprotosis could enhance tumor immunity although cGAS-STING signaling in ccRCC (Figure S9B).

## Discussion

With the advancement of large cohort cancer projects, including TCGA, CPTAC and GEO, and the progress of bioinformatics algorithms, such convenience makes the perception of homotherapy for heteropathy accessible for researchers. ccRCC is the most common subtype of renal cell carcinoma, which is treated as an immunogenic tumor but notorious for its characteristics of immune dysfunction and infiltration of immune inhibitory cells. Even the emergence of checkpoint inhibition has alleviated this condition, and the combination of immune blockade and targeted therapy has been the standard of management for advanced ccRCC patients [39]. The major challenges facing clinical practitioners were that only a substantial proportion of patients could benefit from checkpoint blockade and the extremely horrible adverse reactions of such therapies [40, 41]. Thus, it is urgent to identify new therapeutic targets or adjuvants to aid immune therapy for ccRCC. Growing evidence indicates that copper-induced cell death plays important roles in cell death, while its effects on cancers, especially ccRCC, are still unclear. Copper-induced cell death is a newly discovered mechanism of cell death that is different from apoptosis, ferroptosis, pyroptosis and necroptosis. Past research has focused on the effects of Cu ions or copper ionophores. Yang and Zhang et al. proved that FDX1 was downregulated in various cancers, including ccRCC, and overexpression of FDX1 could repress the malignant tumor phenotype in ccRCC, and vice versa [42, 43]. However, the combined analysis of cuprotosis-related genes and the interaction of cuprotosis and tumor immunity has not been explored. Thus, we described the global characteristics of copper-induced cell death, which might help to guide cancer treatments.

In this study, we performed a comprehensive and systematic analysis of copper-induced cell death-related genes by mining multiomics analysis data in more than 10,000 samples of 33 cancers. We found that copper-induced genes were specifically downregulated in various cancer tissues compared normal tissues, which was correlated with hypermethylation and copy number variation. According to the copper-induced cell death index, ccRCC could be divided into two subtypes, CPCS1 (copper-induced cell death signature scores high groups) and CPCS2 (copper-induced cell death signature scores low groups). The CSP2 subtype shared a higher tumor mutation burden and low tumor stemness index but led to a low ICI therapy response and tumor immunity dysfunction state. Notably, activation of copper-induced cell death may reshape tumor immunity in the ccRCC microenvironment by facilitating the antigen presentation process. In addition, the prognostic model constructed based on subgroup biomarkers exerted satisfactory performance in both the training and validation cohorts. Finally, DLAT, the core gene of copper-induced cell death, could reactivate the copper death pattern and might be a suitable target for ccRCC therapy.

Immunotherapy has been confirmed to be effective in many cancers, including ccRCC. However, therapy resistance diminishes the efficacy of immunotherapy, which can be alleviated by combination therapy, such as targeted therapy and gut microbiota therapy. Among them, copper-induced cell death may collaborate with immunotherapy. Intratumor copper has been shown to influence PD-L1 expression in cancer cells, which suggests that leveraging copper chelation may improve immunotherapy. Wang reported that Cu-triggered hydroxyl radicals and GSH elimination synergized with radiation therapy, remarkably enhancing dendritic cell maturation and promoting antitumour CD8 + T-cell infiltration, thereby potentiating the development of checkpoint blockade immunotherapies against primary and metastatic tumors [44]. Based on copper-induced cell death-related genes, we divided ccRCC into two subtypes, CPCS1 (copper fertile) and CPCS2 (copper desert). The CSP2 subtype exhibited an overall upwards-regulated trend in immune infiltration compared with the CPCS1 subtype. The CSP2 subtype displayed more CD8 + T cells, lacked activated dendritic cells and had reduced DNA damage repair ability, which caused immune dysfunction. The activation of copper-induced death may generate abundant immune antigens for the immune response. There are two ways of copper bonding, including copper chelators and copper ionophores. Copper chelators inhibit cuproplasia, a copper-dependent cellular proliferation, whereas copper ionophores induce cuproptosis. This last term defines copper-dependent cytotoxicity (with a unique mechanism) leading to cell death [45]. Zheng reported that disulfiram/copper codelivery triggered tumor cell autophagy and induced immunogenic cell death, activated tumor-infiltrating macrophages and dendritic cells, and primed T and NK (natural killer) cells, resulting in antitumour immunity and tumor regression [46]. This codelivery system of disulfiram/copper may enhance the immune response of CPCS1 and reactivate the immune antigen effect of CPCS2. Kaur demonstrated that the reticulum-targeting copper (II) complex, a type II immunogenic cell death inducer, elevated intracellular reactive oxygen species (ROS) levels, evoked damage-associated molecular patterns, and promoted breast CSC phagocytosis by macrophages [47]. Therefore, activation of copper-induced death may reshape tumor immunity in the ccRCC microenvironment by regulating the antigen-presenting process and cGAS-STING signaling.

In addition to immunotherapy responses, copper-induced cell death is associated with the dysregulation of many signaling pathways in cancers. Our study indicated that copper-induced cell death-related genes were enriched in RAS-MAPK activation, oxidative phosphorylation accumulation, EMT inhibition and MYC pathways. Consistent with our research, many studies have identified that high copper levels in tumors can regulate kinase activity, inhibit autophagy, and regulate fat metabolism. Pharmacologic copper-chelator treatment using tetrathiomolybdate resulted in decreased tumor burden and increased survival in preclinical murine models of BRAFV600E-driven melanoma [48, 49]. Combination treatment using the BRAF inhibitor vemurafenib together with tetrathiomolybdate enhanced patient survival and alleviated resistance to vemurafenib. This combination was also effective in inducing cell death in melanoma cells resistant to BRAF and MEK1/2 inhibitors. Copper-induced toxicity is inseparable from reactive oxygen species (ROS) induction [50–52]. Elesclomol, a well-known copper ionophore, induces oxidative stress that leads to cancer cell apoptosis. Elesclomol increased copper levels and mitochondrial oxidative stress in the HL-60 leukemic cell line, whereas it did not influence their levels in peripheral blood mononuclear cells. As a prerequisite for tumor metastasis, EMT determines the ability of distant metastasis of tumors. Recent research found that tetrathiomolybdate reduced copper levels and decreased tumor metastasis in triple-negative breast cancer patients by decreasing proliferation, blood vessel formation and mesenchymal transition. MYC is a broadly acting transcription factor that regulates cell differentiation and proliferation through multiple mechanisms, including transcriptional amplification of target genes. Du reported that disulfiram/copper inhibited glycolysis and xenograft growth of GC cells by suppressing the expression of S6K1, c-Myc, and their downstream molecules, including GLUT1, PKM2, and LDHA. Past research has usually focused on the effects of single genes. We divided ccRCC into two distinctive subtypes, which helped to understand alterations in multiple pathways associated with copper-induced death-related genes.

For the root cause, signaling pathway dysregulations often depend on genome mutations. For copper-induced cell death-related genes, we observed that all tested samples had at least one mutation site (729/729). CDKN2A and MTF1 hold the most frequent fractions among mutated genes (54% vs 14%). CDKN2A can form complexes with CDK4 kinase to inhibit cell cycle progression. Mutation or deletion of the CDKN2A gene will relieve the checkpoint function of the cell cycle, and the cells will acquire the ability to proliferate indefinitely. A significant correlation was observed between copper-induced cell death genes and the G2/M checkpoint pathway across cancers. MTF1, a classic metal-sensing transcription factor, promoted myogenesis in response to copper [53]. MTF1 directly bonded to metal-responsive element e within the ATP7B promoter and was considered a strong candidate in regulating ATP7B expression [54]. MTF1 plays an oncogenic role and leads to ovarian cancer metastasis by inducing EMT pathways [55]. In the subtype analysis, CPCS2 had a higher tumor mutation burden than CPCS1, such as mutation of KDM5C, PTEN and XIRP. KDM5C, a histone demethylase gene, could escape from X inactivation and was predominantly mutated in male ccRCC patients. KDM5C was identified to harbor the frameshift mutation in ccRCC with the highest glycogen level. Mutation of KDM5C in ccRCC promoted tumorigenicity by reprogramming glycogen metabolism and inhibiting ferroptosis [56]. It has been found that p53 regulates mitochondrial respiration, where copper depends on mitochondria to function. Seo reported that O-GlcNAcylation of XIAP suppressed colon cancer cell growth and invasion by promoting the proteasomal degradation of O-GlcNAc transferase [57]. The results were confirmed by forest analysis. For example, whole-exome sequencing detected the somatically mutated gene DNAH11 in RCC samples with PD-L1-positive expression [58]. Verma reported that the rs2285947 variant of the DNAH11 gene predicted poor prognosis for ovarian and breast cancer patients [59]. For copy number variations, the CPCS2 subtype presented higher CNV amplification and deletion frequencies than the CPCS1 subtype on chromosomes. Some copper-induced cell death-related genes were contained in these mutated regions, such as CDKN2B and FGFR4. Consistent with our study, Fernandes found that the most significant copy number alterations of ccRCC were loss of 3p (87.3%), 14q (35.8%), 6q (29.3%), 9p (28.6%) and 10q (25.0%) and gains of 5q (59.7%), 7p (29.3%) and 16q (20.6%). Genes mapping to CNA significant regions included SETD2, BAP1, FLT4, PTEN, FGFR4 and NSD1 [60]. FGFR4 regulates tumor subtype differentiation and induces metastatic disease in breast cancer [61]. Therefore, copper-induced death subtyping, at least in part, explains tumor heterogeneity through the regulation of various signaling pathways in ccRCC.

Based on multiomics data, our study provided clues for choosing clinical treatment options. The drug sensitivity profiles were investigated between subgroups and their matched cell lines. There were certain differences in the effective targets of CPCS1 and CPCS2 subtypes, which were VEGFR/PDGFR/MET and EGFR/SRC, respectively. In the era of targeted therapy and immunotherapy, the effectiveness of these targets can provide certain clinical guidance for immunotherapy. The CellMiner database provides potential therapeutic targets for the copper-induced death subtype. For instance, although CPCS2 was less sensitive to many molecular inhibitors, the ROCK inhibitor GSK269962A displayed a unique curative effect on copper-induced death desert type (CPCS2). ccRCC is often resistant to chemotherapy. Our data suggested that the combination of paclitaxel and immunotherapy may reactivate the responses of the CPCS2 subtype to paclitaxel. Elesclomol was able to target resistant cancer cells, including cisplatin and proteasome inhibitor resistance. Antiapoptotic inhibitors of the BCL-2 family synergize with tetrathiomolybdate to induce apoptosis in melanoma cells resistant to BRAF and MEK1/2 inhibitors [62]. Numerous studies have suggested that copper transporters contribute to cisplatin resistance by controlling its uptake and export from tumor cells. Recent studies demonstrated that deletion of ATP7A, a copper pump, increased cisplatin sensitivity and limited tumor growth in mice [63]. Molecular profiling based on copper-induced death could lead to the development of more individualized therapeutic targets.

Furthermore, the best risk model RCC-CUPT4 was established using Cox regression analysis. The four most relevant genes were MGAM, PTPRB, PAGE2B and RTL1. A high RCC-CUPT4 score indicated poor outcomes of ccRCC. Previous studies have shown that MGAM is closely related to cancer immunotherapy responsiveness in non-small cell lung cancer [64]. Recent studies have shown that PTPRB promotes colon cancer invasion and metastasis by inducing EMT, and PTPRB is a potential therapeutic target for colon cancer [65]. Riordan et al. found that RTL1 was an important oncogene in hepatocarcinogenesis [66]. The high sensitivity and specificity of the RCC-CUPT4 risk model was further identified in both the TCGA-ccRCC and JAPAN-KIRC databases. By analysing the important fraction of copper-induced cell death, we found that DLAT may play a core role in copper biology. The AUC of the DLAT prediction model achieved 1.00 in Zhao glioblastoma (PD-1) and 0.80 in Nathanson melanoma (CTLA4) [67]. In vitro and in vivo experiments suggested that DLAT effectively inhibited the growth and metastasis of renal cancer. Faqihi reported that radiation-induced blockade of autophagic flux stimulated redirection of DLAT to the cell surface via a noncanonical secretory autophagy pathway [68]. Such trafficked membrane proteins could provide a unique pool for therapeutic drug delivery. DLAT may be a suitable target to induce the copper death pattern, as indicated by the upregulation of copper and downregulation of DLAT. The mechanism of DLAT has rarely been studied, and further research is warranted.

Although our study conducted genomics analysis and cell line experiments of copper-induced death, there are certain limitations. Our main findings were confirmed by bioinformatics analysis, which requires further experimental validation. The drug susceptibility of the two subtypes was different, but it also needs to be verified by subsequent animal and clinical experiments. More importantly, the prognostic model may be affected by some confounding factors, and more datasets are needed for revision and refinement.

In conclusion, prior to our study, a few reports identified molecular subtypes of ccRCC based on gene expression profiles or mutational signatures [32, 69–71]. To the best of our knowledge, this is the first study to systematically analyse the roles of cuprotosis-related genes in multiple cancers and to identify two molecular subtypes of ccRCC. Activation of cuprotosis might function as a treatment approach among multiple cancers. Such a signature could reshape tumor immunity in the ccRCC microenvironment by activating antigen presentation and cGAS-STING signaling. The upregulation of DLAT expression in ccRCC cell lines could reactivate the copper death pattern and be treated as a suitable target for ccRCC therapy. Our study provides a new reference for understanding the function of copper-induced death in cancers, which may provide clinical guidance for ccRCC treatment.

## Supplementary Information

**Additional file 1: Figure S1.** Workflow of this study. **Figure S2.** Copper-induced cell death-related genes are dysregulated in multiple cancers. (A) The expression of FDX1 in cancer and normal tissues. (B) Mutation frequencies of copper-induced cell death-related genes in multiple cancers. (C) The correlations between overall survival and methylation levels of copper-induced cell death-related genes. **Figure S3.** The drug sensitivity assessment of several copper-induced cell death-related genes to molecular inhibitors in cancer cell lines. **Figure S4.** Pathway enrichment analysis of ccRCC subtypes. (A) GSEA pathway analysis of differentially expressed genes between the two subtypes. Heatmap of tumor microenvironment-related pathways (B) and metabolism-related pathway (C) enrichment scores between the two subtypes. **Figure S5.** Correlation of copper-induced cell death-related gene expression and IC50 of tested drugs obtained from the CellMiner and GDSC databases (A) Drug sensitivity values in the form of normalized AUC using the GDSC renal cancer cell database. (B) Drug sensitivity values in the form of normalized AUC using the CCLE database. **Figure S6.** Verification of the copper-induced death-related risk model in the JAPAN-KIRC cohort. (A) Risk score analysis of patients in the JAPAN-KIRC cohort. (B) Survival analysis for OS of the two risk signatures in the JAPAN-KIRC cohort. (C) The time-dependent ROC curves for the two risk signatures in the JAPAN-KIRC cohort. **Figure S7.** The functional verification of DLAT. (A) The expression of DLAT in renal cancer and normal tissues. (B) The expression of DLAT in renal cancer of different stages. (C) Immunohistochemical score of DLAT in cancer and normal kidney tissues. (D) Survival analysis for PFS of DLAT expression. (E) The association between DLAT mutation and immune cell infiltration in ccRCC. (F) The association between DLAT expression and immune signatures across cancers. **Figure S8.** Different fluorescence and Edu level, and downregulated cuproptosis state in ccRCC malignant cells. (A)Different fluorescence and DdU positive cells (B) between NC and cuproptosis inducer treated groups, respectively. (C, D) Umap showing the cuproptosis score at the single-cell level for ccRCC. **Figure S9.** Impact of cuproptosis in tumor immunity of ccRCC. (A) Correlation of cuproptosis and immune infiltration degree among TCGA-KIRC, JAPAN-KIRC and Cancer cell-KIRC cohorts. (B) Schematic diagram of the activation of tumor immunity in ccRCC through cuproptosis.

**Additional file 2: Table S1.** List of copper-induced cell death-related genes. **Table S2**. Clinicopathological features of different subtypes in ccRCC.

**Additional file 3: Table S3.** Recurrent amplification and deletion regions in the two subtypes calculated by GISTIC2.0.

**Additional file 4: Table S4.** List of 138 kinds of small-molecule drugs that could be used as potential drugs for ccRCC.

## Data Availability

All data generated or analysed during this study are included in this published article and its supplementary information files.
