# Supplementary material for: A new thinking: deciphering the aberrance and clinical implication of copper-death signatures in clear cell renal cell carcinoma
Source: Cell Biosci. 2022 Dec 29;12:209. doi: 10.1186/s13578-022-00948-7 (PMC9801655; doi:10.1186/s13578-022-00948-7)
Supplement: Supplementary file 1 — Additional file 1: Figure S1. Workflow of this study. Figure S2. Copper-induced cell death-related genes are dysregulated in multiple cancers. (A) The expression of FDX1 in cancer and normal tissues. (B) Mutation frequencies of copper-induced cell death-related genes in multiple cancers. (C) The correlations between overall survival and methylation levels of copper-induced cell death-related genes. Figure S3. The drug sensitivity assessment of several copper-induced cell death-related genes to molecular inhibitors in cancer cell lines. Figure S4. Pathway enrichment analysis of ccRCC subtypes. (A) GSEA pathway analysis of differentially expressed genes between the two subtypes. Heatmap of tumor microenvironment-related pathways (B) and metabolism-related pathway (C) enrichment scores between the two subtypes. Figure S5. Correlation of copper-induced cell death-related gene expression and IC50 of tested drugs obtained from the CellMiner and GDSC databases (A) Drug sensitivity values in the form of normalized AUC using the GDSC renal cancer cell database. (B) Drug sensitivity values in the form of normalized AUC using the CCLE database. Figure S6. Verification of the copper-induced death-related risk model in the JAPAN-KIRC cohort. (A) Risk score analysis of patients in the JAPAN-KIRC cohort. (B) Survival analysis for OS of the two risk signatures in the JAPAN-KIRC cohort. (C) The time-dependent ROC curves for the two risk signatures in the JAPAN-KIRC cohort. Figure S7. The functional verification of DLAT. (A) The expression of DLAT in renal cancer and normal tissues. (B) The expression of DLAT in renal cancer of different stages. (C) Immunohistochemical score of DLAT in cancer and normal kidney tissues. (D) Survival analysis for PFS of DLAT expression. (E) The association between DLAT mutation and immune cell infiltration in ccRCC. (F) The association between DLAT expression and immune signatures across cancers. Figure S8. Different fluorescence and Edu level, and dow [file 13578_2022_948_MOESM1_ESM.pdf]

Figure S1

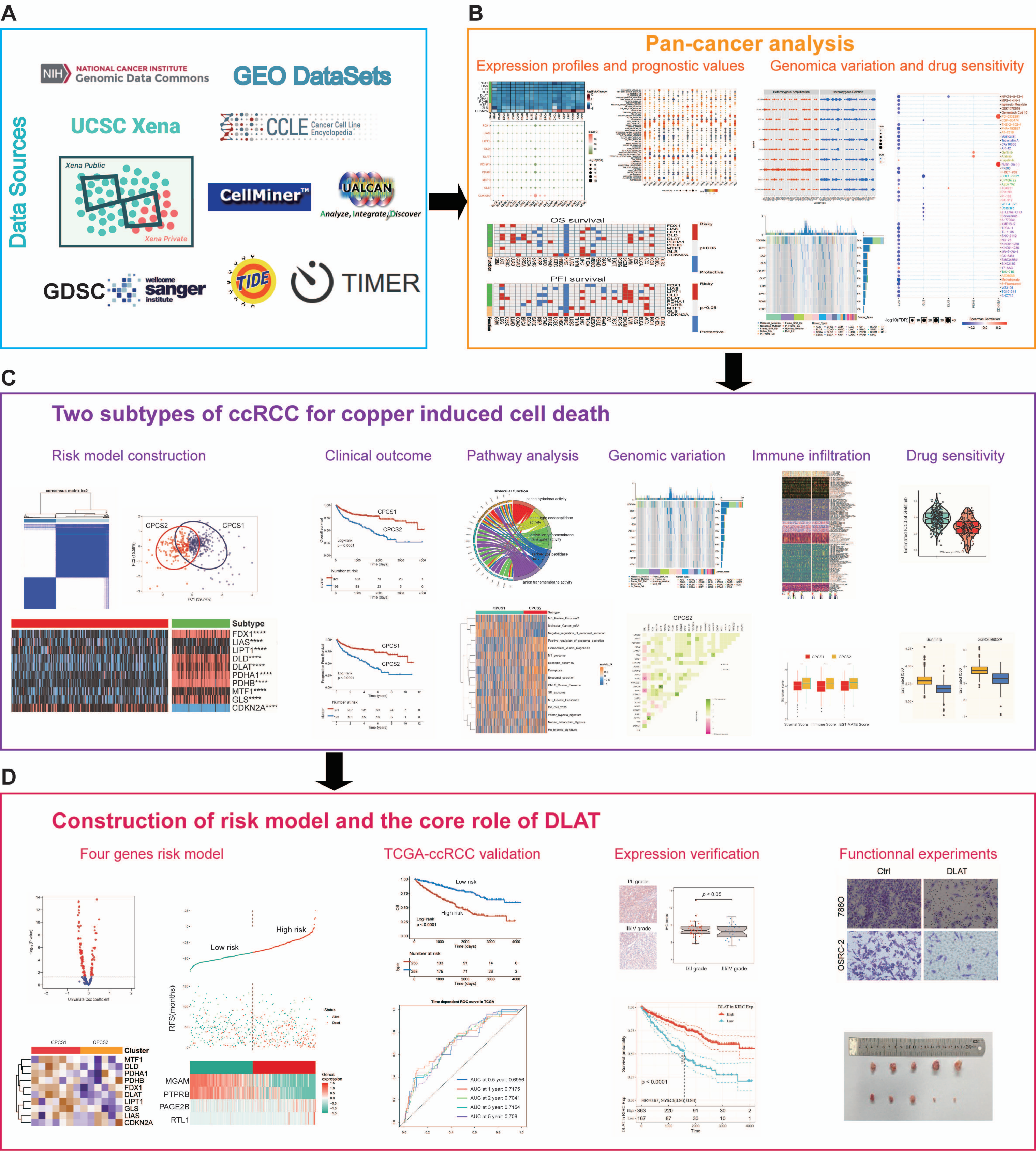
A
Data Sources
NIH NATIONAL CANCER INSTITUTE Genomic Data Commons
GEO DataSets
UCSC Xena
CCLE Cancer Cell Line Encyclopedia
Xena Public
Xena Private
CellMiner™
UALCAN
Analyze, Integrate, Discover
GDSC
wellcome sanger institute
TIDE
TIMER
B
Pan-cancer analysis
Expression profiles and prognostic values
Genomica variation and drug sensitivity
OS survival
PFI survival
Risky
p>0.05
Protective
C
Two subtypes of ccRCC for copper induced cell death
Risk model construction
Clinical outcome
Pathway analysis
Genomic variation
Immune infiltration
Drug sensitivity
consensus matrix k=2
CPCS2
CPCS1
Subtype
FDX1****
LIAS****
LIPT1****
DLD****
DLAT****
PDHA1****
PDHB****
MTF1****
GLS****
CDKN2A****
Molecular function
CPCS2
D
Construction of risk model and the core role of DLAT
Four genes risk model
TCGA-ccRCC validation
Expression verification
Functionnal experiments
Low risk
High risk
RFS(months)
MGAM
PTPRB
PAGE2B
RTL1
Cluster
MTF1
DLD
PDHA1
PDHB
FDX1
DLAT
LIPT1
GLS
LIAS
CDKN2A
Log-rank
p < 0.0001
Number at risk
AUC at 0.5 year: 0.6956
AUC at 1 year: 0.7175
AUC at 2 year: 0.7041
AUC at 3 year: 0.7154
AUC at 5 year: 0.708
I/II grade
III/IV grade
p < 0.05
DLAT in KIRC Exp
High
Low
p < 0.0001
HR=0.97, 95%CI(0.96, 0.98)
Ctrl
DLAT
786O
OSRC-2

Figure S2

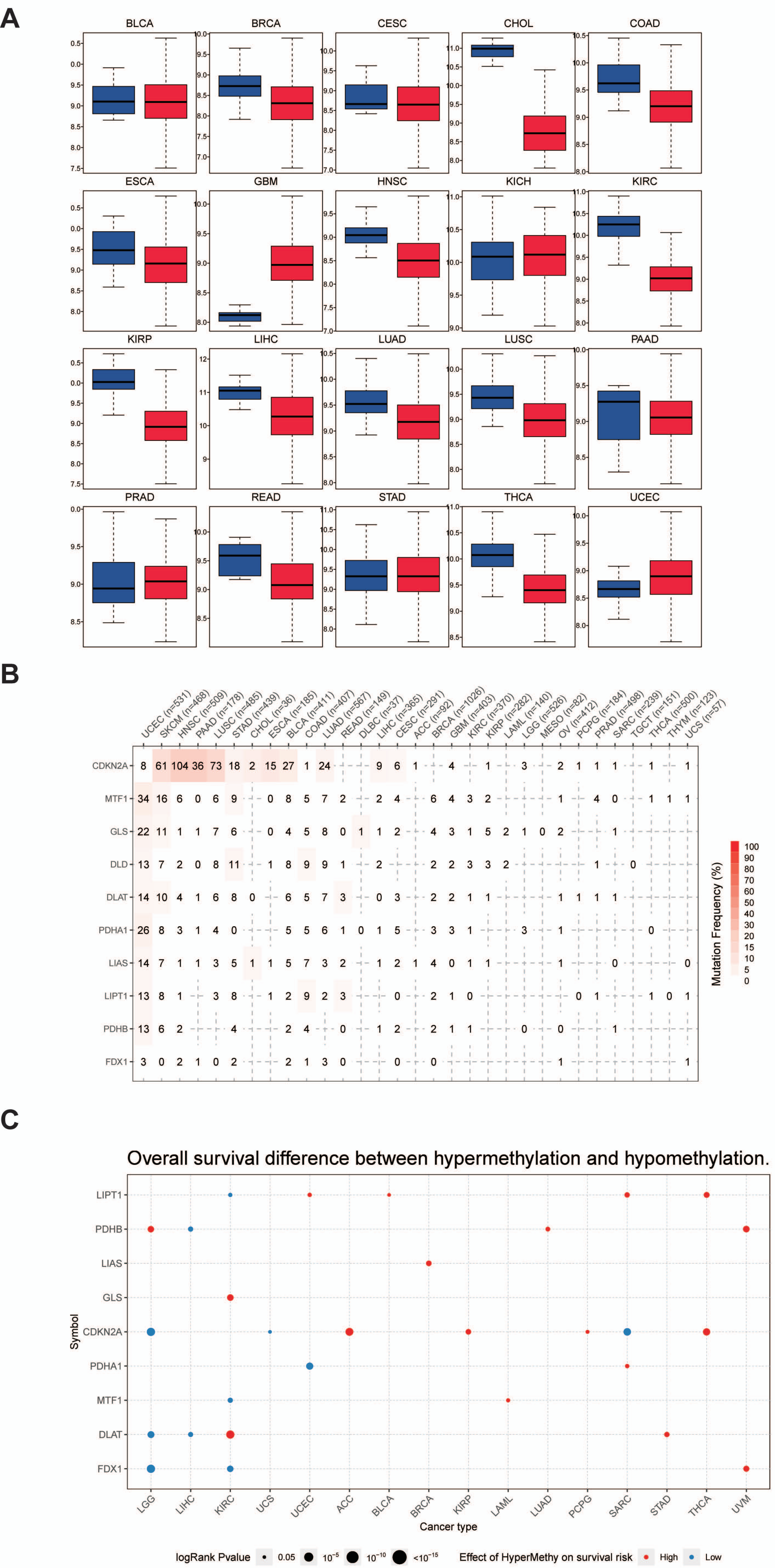

A
BLCA
BRCA
CESC
CHOL
COAD
ESCA
GBM
HNSC
KICH
KIRC
KIRP
LIHC
LUAD
LUSC
PAAD
PRAD
READ
STAD
THCA
UCEC
B
UCEC (n=531) SKCM (n=468) HNSC (n=509) PAAD (n=178) LUSC (n=485) STAD (n=439) CHOL (n=36) ESCA (n=185) BLCA (n=411) COAD (n=407) LUAD (n=567) READ (n=149) DLBC (n=37) LIHC (n=365) CESC (n=291) ACC (n=92) BRCA (n=1026) GBM (n=403) KIRC (n=370) KIRP (n=282) LAML (n=140) LGG (n=526) MESO (n=82) OV (n=412) PCPG (n=184) PRAD (n=498) SARC (n=239) TGCT (n=151) THCA (n=500) THYM (n=123) UCS (n=57)
CDKN2A
MTF1
GLS
DLD
DLAT
PDHA1
LIAS
LIPT1
PDHB
FDX1
Mutation Frequency (%)
C
Overall survival difference between hypermethylation and hypomethylation.
LIPT1
PDHB
LIAS
GLS
CDKN2A
PDHA1
MTF1
DLAT
FDX1
Symbol
LGG LIHC KIRC UCS UCEC ACC BLCA BRCA KIRP LAML LUAD PCPG SARC STAD THCA UVM
Cancer type
logRank Pvalue 0.05 10⁻⁵ 10⁻¹⁰ <10⁻¹⁵
Effect of HyperMethy on survival risk High Low

Figure S3

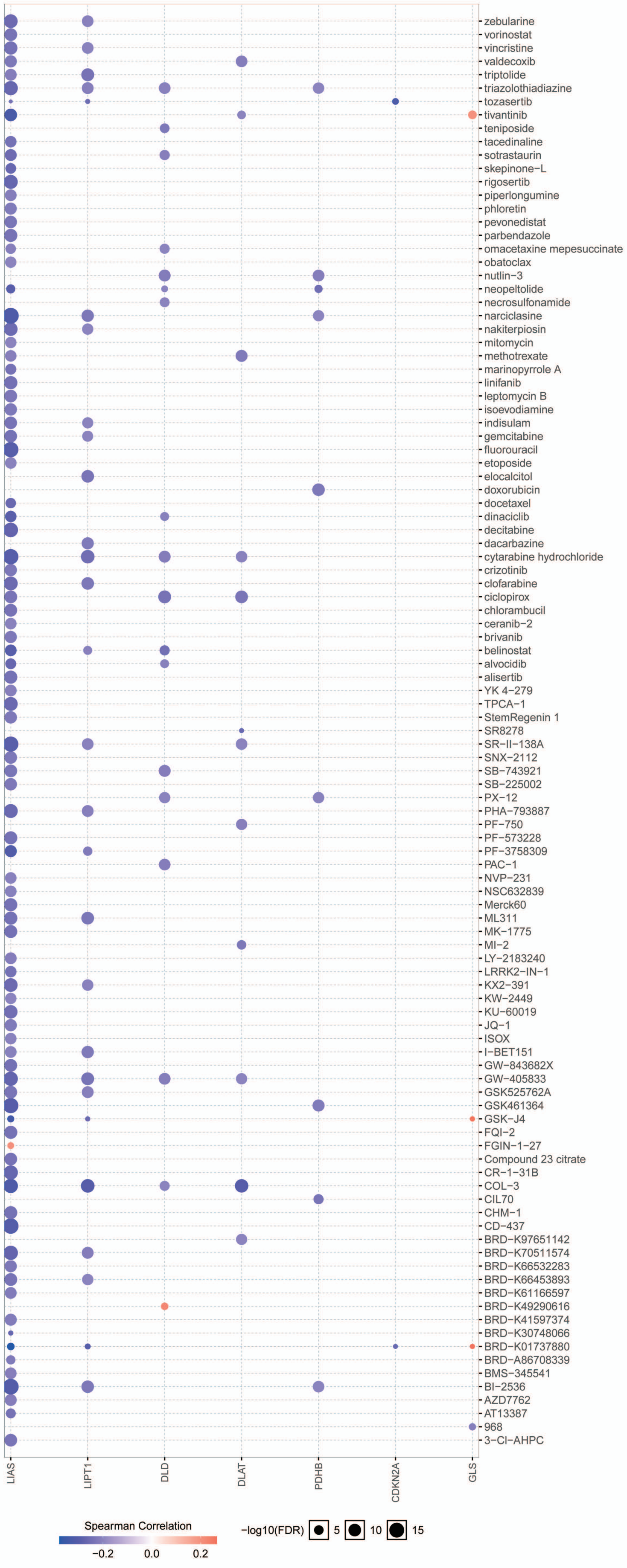
zebularine
vorinostat
vincristine
valdecoxib
triptolide
triazolothiadiazine
tozasertib
tivantinib
teniposide
tacedinaline
sotrastaurin
skepinone−L
rigosertib
piperlongumine
phloretin
pevonedistat
parbendazole
omacetaxine mepesuccinate
obatoclax
nutlin−3
neopeltolide
necrosulfonamide
narciclasine
nakiterpiosin
mitomycin
methotrexate
marinopyrrole A
linifanib
leptomycin B
isoevodiamine
indisulam
gemcitabine
fluorouracil
etoposide
elocalcitol
doxorubicin
docetaxel
dinaciclib
decitabine
dacarbazine
cytarabine hydrochloride
crizotinib
clofarabine
ciclopirox
chlorambucil
ceranib−2
brivanib
belinostat
alvocidib
alisertib
YK 4−279
TPCA−1
StemRegenin 1
SR8278
SR−II−138A
SNX−2112
SB−743921
SB−225002
PX−12
PHA−793887
PF−750
PF−573228
PF−3758309
PAC−1
NVP−231
NSC632839
Merck60
ML311
MK−1775
MI−2
LY−2183240
LRRK2−IN−1
KX2−391
KW−2449
KU−60019
JQ−1
ISOX
I−BET151
GW−843682X
GW−405833
GSK525762A
GSK461364
GSK−J4
FQI−2
FGIN−1−27
Compound 23 citrate
CR−1−31B
COL−3
CIL70
CHM−1
CD−437
BRD−K97651142
BRD−K70511574
BRD−K66532283
BRD−K66453893
BRD−K61166597
BRD−K49290616
BRD−K41597374
BRD−K30748066
BRD−K01737880
BRD−A86708339
BMS−345541
BI−2536
AZD7762
AT13387
968
3−Cl−AHPC
LIAS
LIPT1
DLD
DLAT
PDHB
CDKN2A
GLS
Spearman Correlation
−0.2
0.0
0.2
−log10(FDR)
5
10
15

Figure S4

A

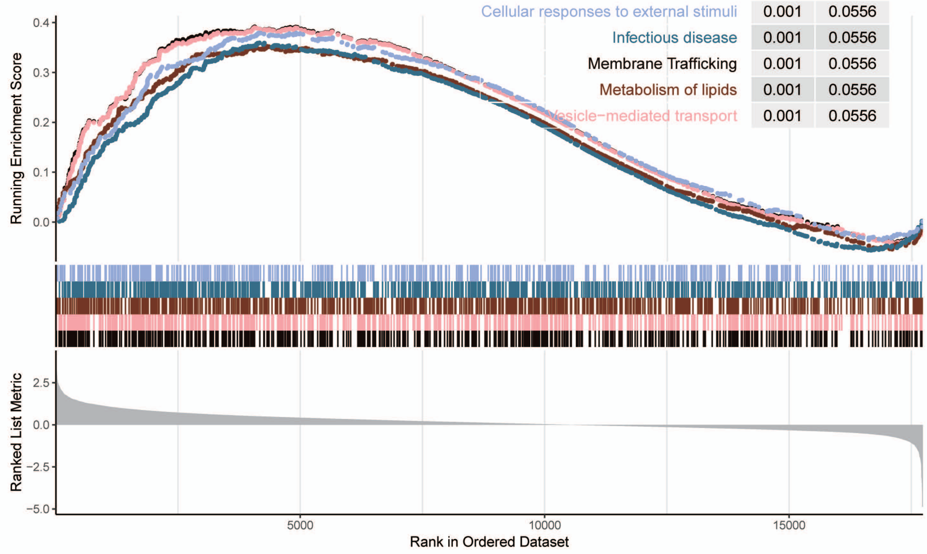

B

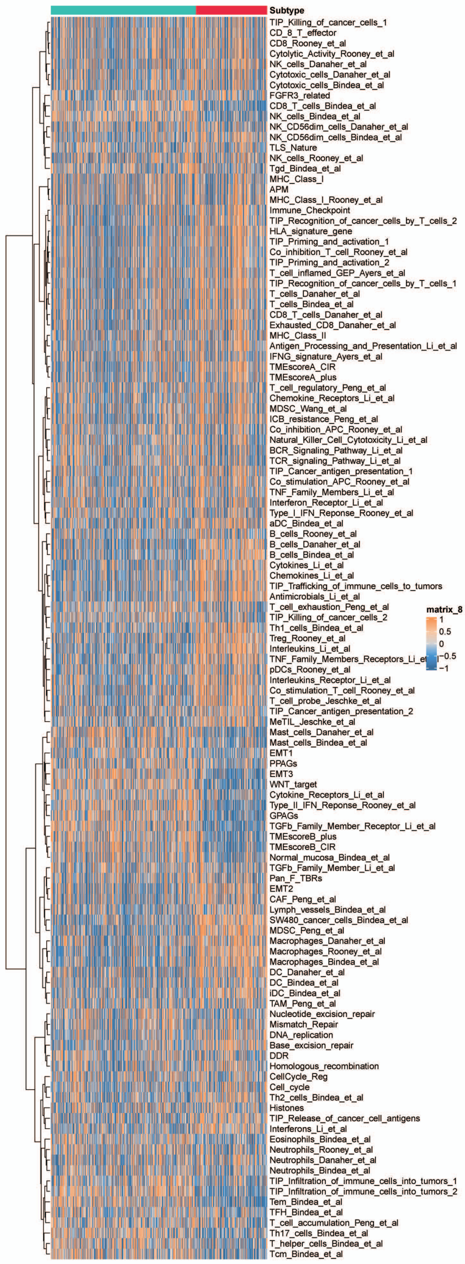

C

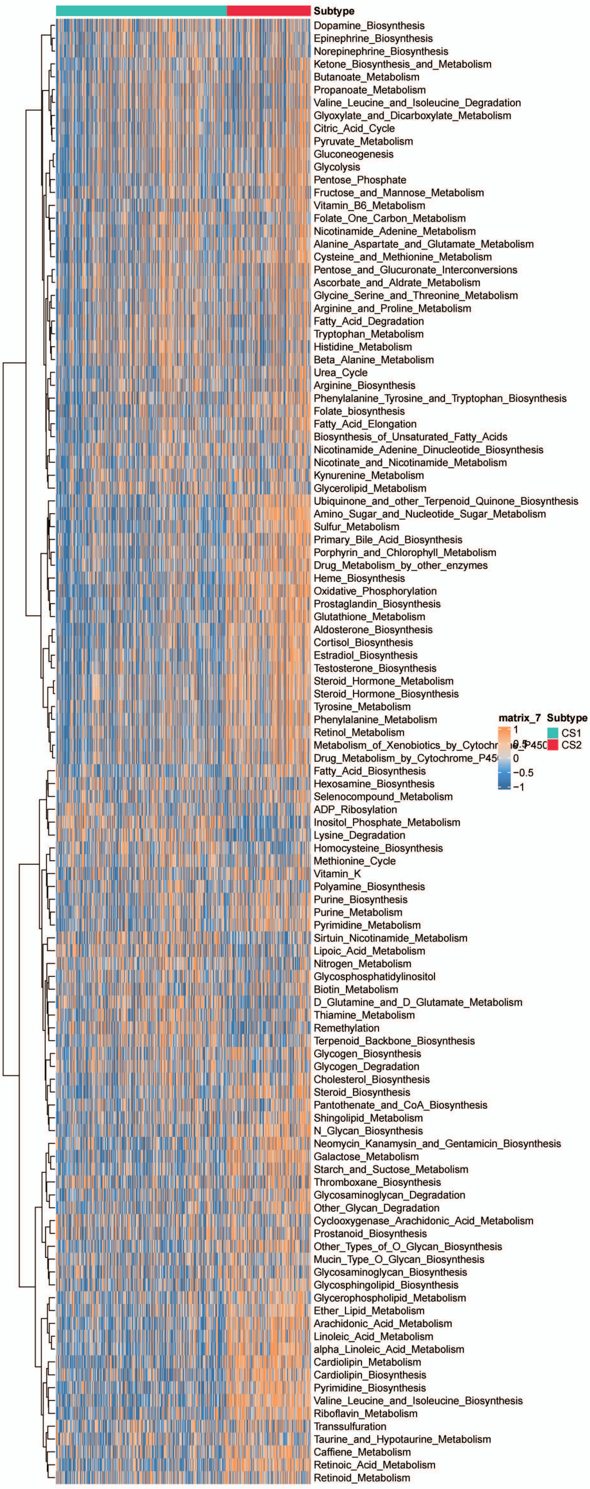

Figure S5

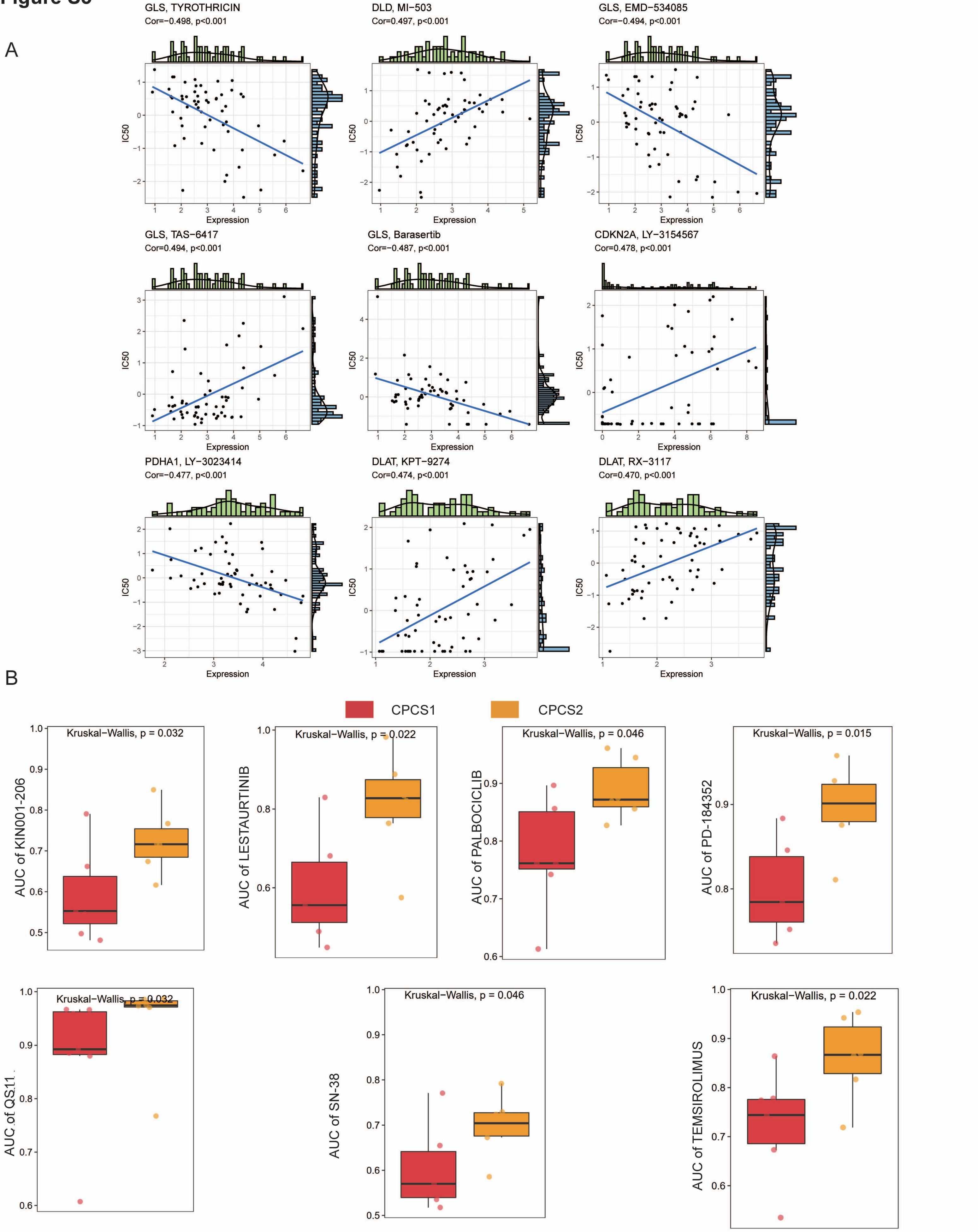
A
GLS, TYROTHRICIN
Cor=−0.498, p<0.001
DLD, MI−503
Cor=0.497, p<0.001
GLS, EMD−534085
Cor=−0.494, p<0.001
GLS, TAS−6417
Cor=0.494, p<0.001
GLS, Barasertib
Cor=−0.487, p<0.001
CDKN2A, LY−3154567
Cor=0.478, p<0.001
PDHA1, LY−3023414
Cor=−0.477, p<0.001
DLAT, KPT−9274
Cor=0.474, p<0.001
DLAT, RX−3117
Cor=0.470, p<0.001
IC50
Expression
B
CPCS1
CPCS2
Kruskal−Wallis, p = 0.032
AUC of KIN001-206
Kruskal−Wallis, p = 0.022
AUC of LESTAURTINIB
Kruskal−Wallis, p = 0.046
AUC of PALBOCICLIB
Kruskal−Wallis, p = 0.015
AUC of PD-184352
Kruskal−Wallis, p = 0.032
AUC of QS11
Kruskal−Wallis, p = 0.046
AUC of SN-38
Kruskal−Wallis, p = 0.022
AUC of TEMSIROLIMUS

Figure S6

A

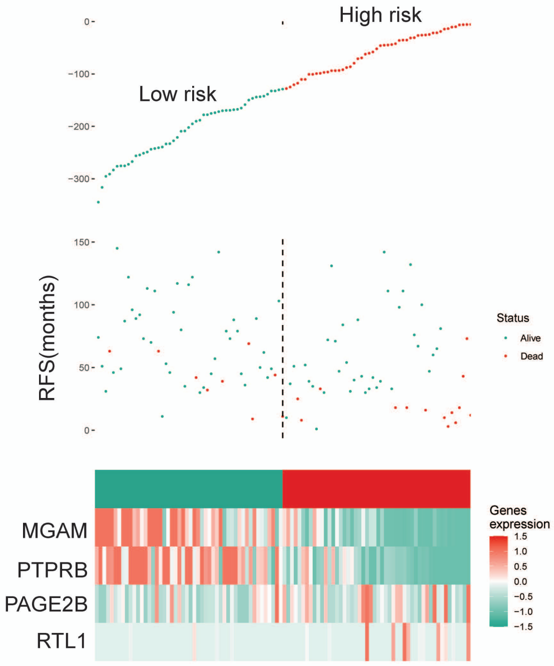

B

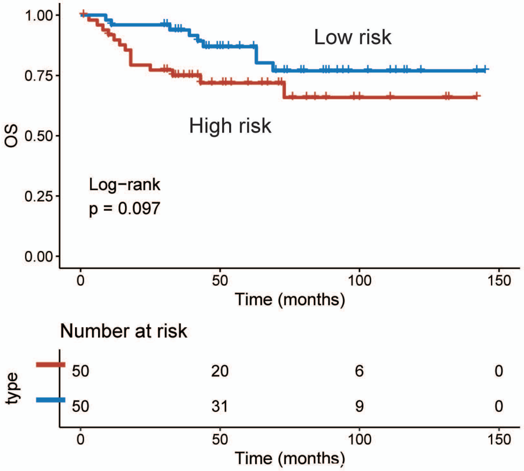

C

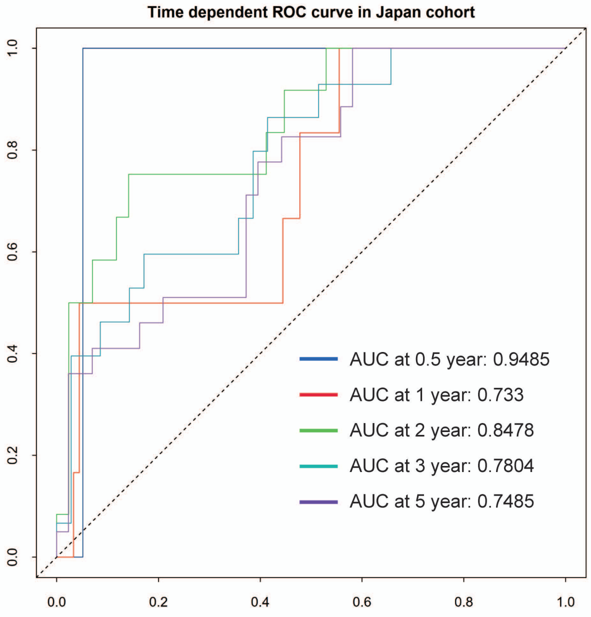

Figure S7

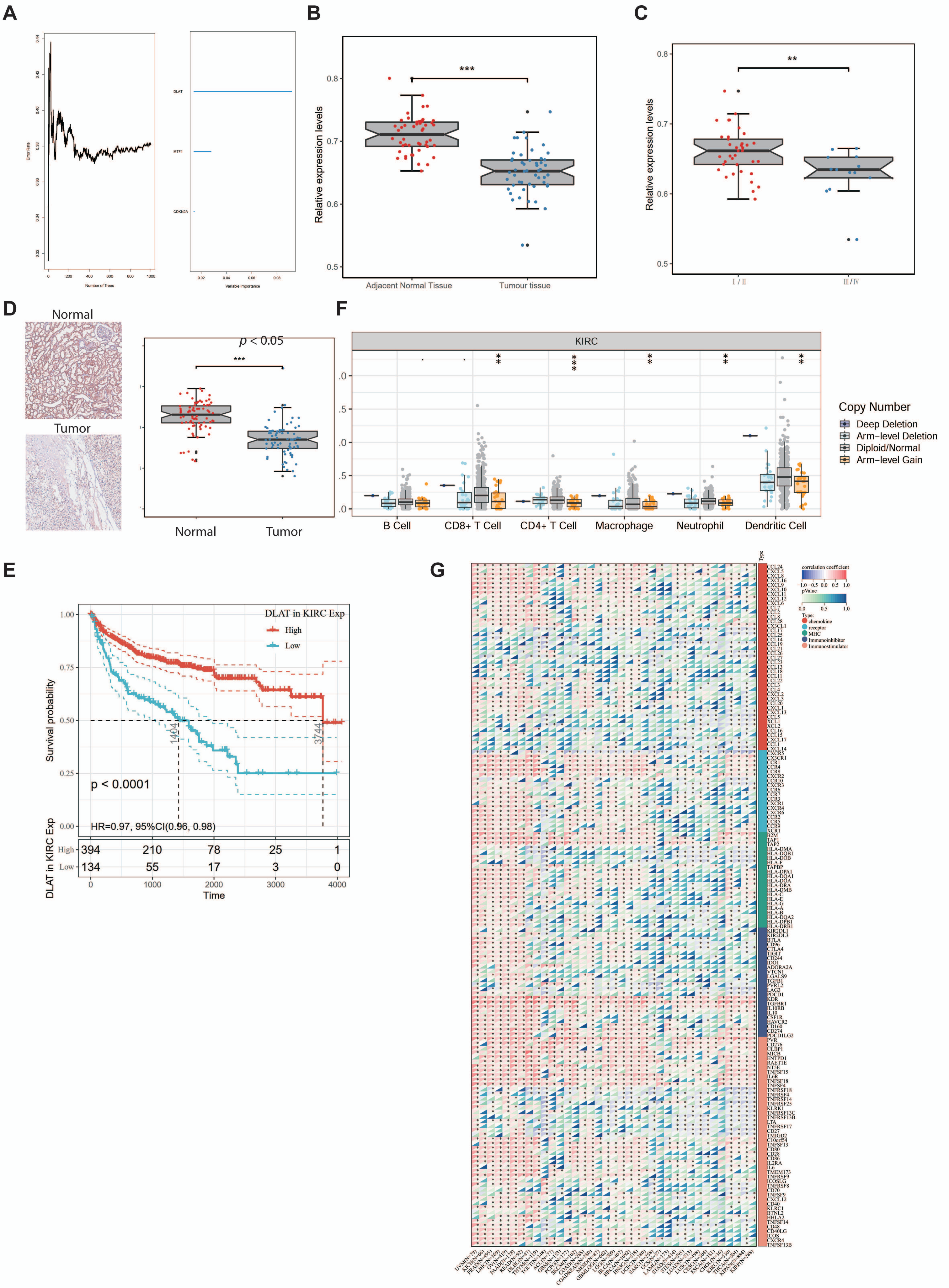

A
Error Rate
Number of Trees
DLAT
MTF1
CDKN2A
Variable Importance
B
***
Relative expression levels
Adjacent Normal Tissue
Tumour tissue
C
**
Relative expression levels
Ⅰ/Ⅱ
Ⅲ/Ⅳ
D
Normal
Tumor
p < 0.05
***
Normal
Tumor
F
KIRC
B Cell
CD8+ T Cell
CD4+ T Cell
Macrophage
Neutrophil
Dendritic Cell
Copy Number
Deep Deletion
Arm−level Deletion
Diploid/Normal
Arm−level Gain
E
DLAT in KIRC Exp
High
Low
Survival probability
p < 0.0001
HR=0.97, 95%CI(0.96, 0.98)
1404
3744
DLAT in KIRC Exp
High 394 210 78 25 1
Low 134 55 17 3 0
Time
G
Type
correlation coefficient
pValue
Type:
chemokine
receptor
MHC
Immunoinhibitor
Immunostimulator

Figure S8

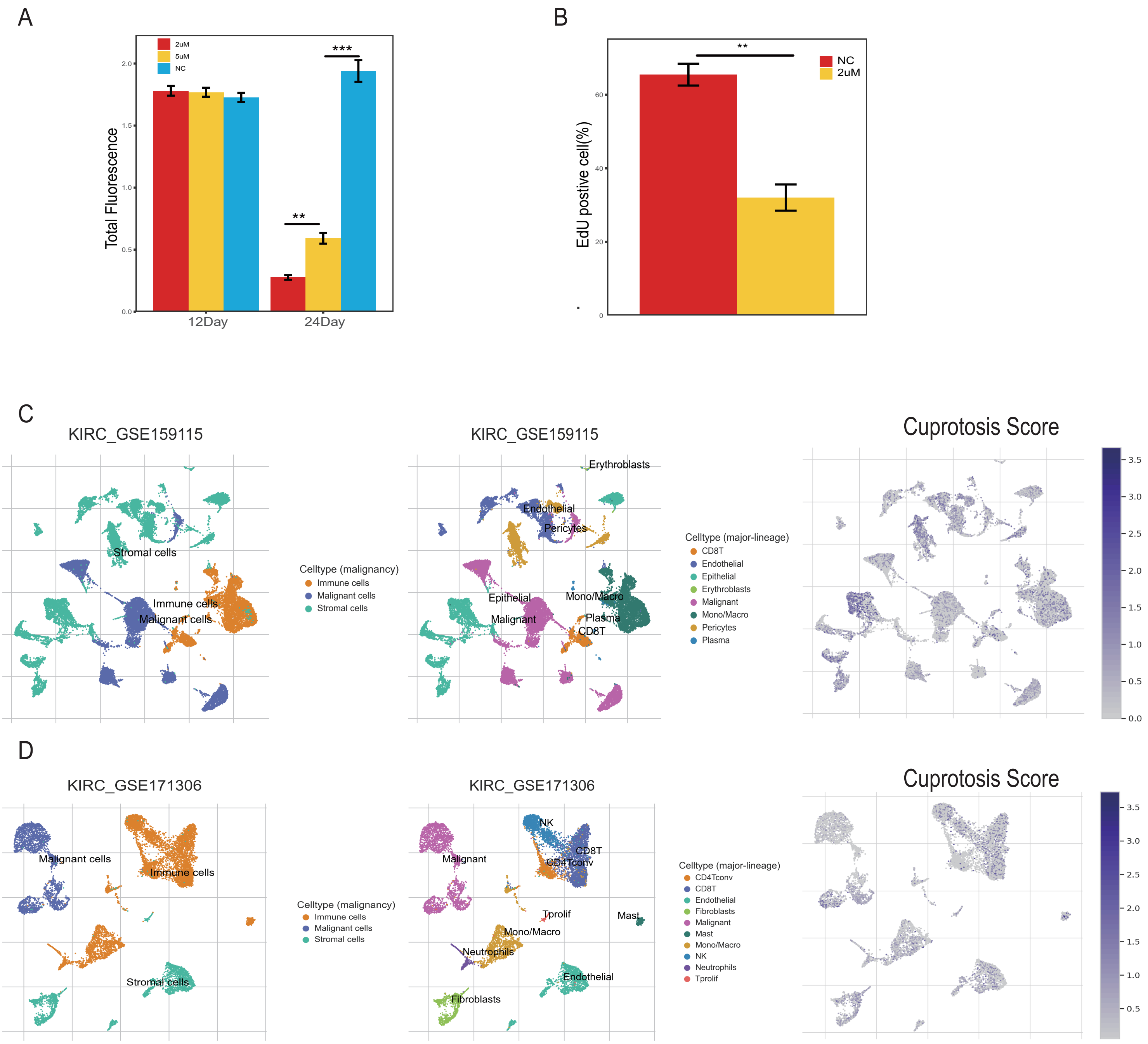
A
2uM
5uM
NC
Total Fluorescence
2.0
1.5
1.0
0.5
0.0
***
**
12Day
24Day
B
NC
2uM
**
EdU postive cell(%)
60
40
20
0
C
KIRC_GSE159115
Stromal cells
Immune cells
Malignant cells
Celltype (malignancy)
Immune cells
Malignant cells
Stromal cells
KIRC_GSE159115
Erythroblasts
Endothelial
Pericytes
Epithelial
Malignant
Mono/Macro
Plasma
CD8T
Celltype (major-lineage)
CD8T
Endothelial
Epithelial
Erythroblasts
Malignant
Mono/Macro
Pericytes
Plasma
Cuprotosis Score
3.5
3.0
2.5
2.0
1.5
1.0
0.5
0.0
D
KIRC_GSE171306
Malignant cells
Immune cells
Stromal cells
Celltype (malignancy)
Immune cells
Malignant cells
Stromal cells
KIRC_GSE171306
NK
CD8T
CD4Tconv
Malignant
Tprolif
Mast
Mono/Macro
Neutrophils
Endothelial
Fibroblasts
Celltype (major-lineage)
CD4Tconv
CD8T
Endothelial
Fibroblasts
Malignant
Mast
Mono/Macro
NK
Neutrophils
Tprolif
Cuprotosis Score
3.5
3.0
2.5
2.0
1.5
1.0
0.5

Figure S9

A

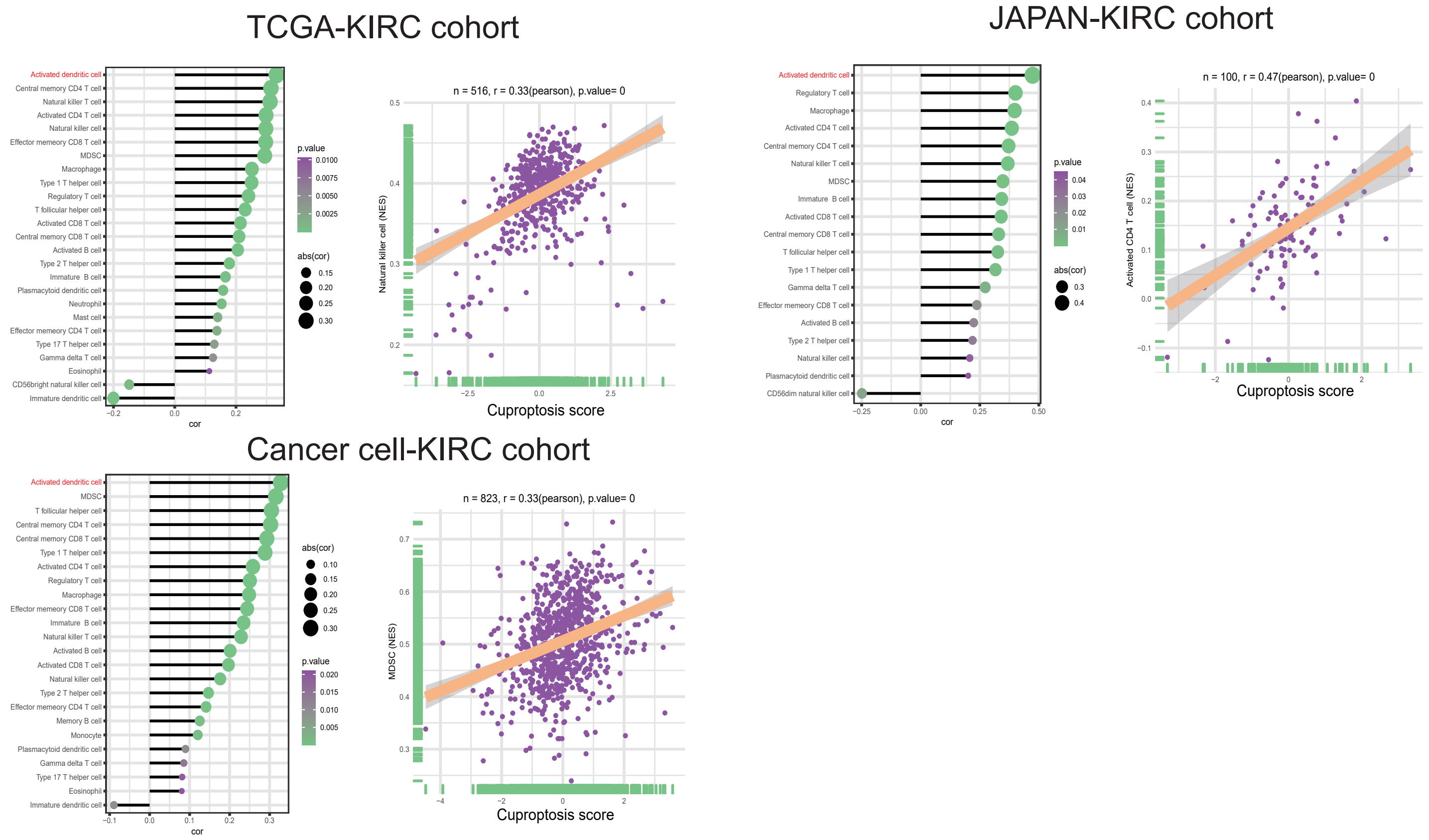

TCGA-KIRC cohort
JAPAN-KIRC cohort
Cancer cell-KIRC cohort
n = 516, r = 0.33(pearson), p.value= 0
n = 100, r = 0.47(pearson), p.value= 0
n = 823, r = 0.33(pearson), p.value= 0
Natural killer cell (NES)
Activated CD4 T cell (NES)
MDSC (NES)
Cuproptosis score
p.value
abs(cor)
cor
Activated dendritic cell

B

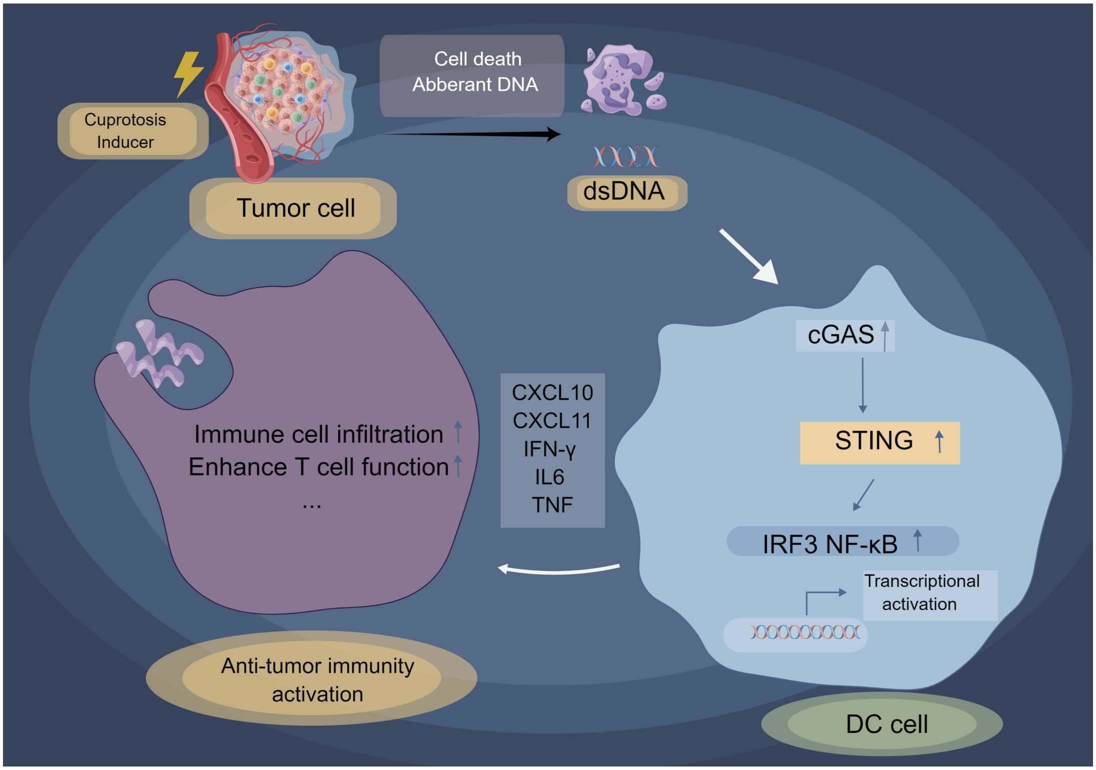

Cuprotosis Inducer
Tumor cell
Cell death Abberant DNA
dsDNA
cGAS
STING
IRF3 NF-κB
Transcriptional activation
CXCL10
CXCL11
IFN-γ
IL6
TNF
Immune cell infiltration
Enhance T cell function
...
Anti-tumor immunity activation
DC cell
